# d-PET-controlled “off-on” Polarity-sensitive Probes for Reporting Local Hydrophilicity within Lysosomes

**DOI:** 10.1038/srep35627

**Published:** 2016-10-21

**Authors:** Hao Zhu, Jiangli Fan, Huiying Mu, Tao Zhu, Zhen Zhang, Jianjun Du, Xiaojun Peng

**Affiliations:** 1 State Key Laboratory of Fine Chemicals, Dalian University of Technology, 2 Linggong Road, Dalian, 116024, China

## Abstract

Polarity-sensitive fluorescent probes are powerful chemical tools for studying biomolecular structures and activities both *in vitro* and *in vivo*. However, the lack of “off-on” polarity-sensing probes has limited the accurate monitoring of biological processes that involve an increase in local hydrophilicity. Here, we design and synthesize a series of “off-on” polarity-sensitive fluorescent probes **BP** series consisting of the difluoroboron dippyomethene (BODIPY) fluorophore connected to a quaternary ammonium moiety *via* different carbon linkers. All these probes showed low fluorescence quantum yields in nonpolar solution but became highly fluorescent in polar media. **BP-2**, which contains a two-carbon linker and a trimethyl quaternary ammonium, displayed a fluorescence intensity and quantum yield that were both linearly correlated with solvent polarity. In addition, **BP-2** exhibited high sensitivity and selectivity for polarity over other environmental factors and a variety of biologically relevant species. **BP-2** can be synthesized readily *via* an unusual Mannich reaction followed by methylation. Using electrochemistry combined with theoretical calculations, we demonstrated that the “off-on” sensing behavior of **BP-2** is primarily due to the polarity-dependent donor-excited photoinduced electron transfer (d-PET) effect. Live-cell imaging established that **BP-2** enables the detection of local hydrophilicity within lysosomes under conditions of lysosomal dysfunction.

Polarity is an important environmental parameter that reflects the hydrophobicity or hydrophilicity of local environment. In biological systems, especially at the cellular level, polarity determines the interaction activities of a large number of proteins and controls the permeability of membrane compartments. However, polarity is a complex factor and encompasses a range of non–covalent interactions, including dipolarity/polarizability and hydrogen bonding^1^,^2^. Thus, realising its measurement in a straightforward manner in live cells is difficult, necessitating the development of new tools.

Polarity-sensitive fluorescent probes are powerful chemical tools for studying biomolecular structures and activities both *in vitro* and *in vivo*^3^,^4^,^5^,^6^,^7^,^8^,^9^,^10^,^11^,^12^,^13^,^14^. Numerous efforts have been devoted to the development of intramolecular-charge-transfer (ICT)-based probes for polarity sensing. They typically exhibit a marked red shift in their emission spectra and/or a decrease in their fluorescence intensities upon detecting an increase in surrounding polarity (“on-off” type). When conjugated to specific tags, such as amino acids^4^,^5^,^6^ and aliphatic chains^7^,^8^,^9^, these probes have been successfully applied in the study of structures and activities of hydrophobic proteins and membranes. However, the lack of “off-on” polarity-sensitive probes has limited the accurate monitoring of biological processes that involve an increase in local hydrophilicity, e.g., protein unfolding. Acridine^15^, pyrene-3-carboxaldehyde (PCA)^16^, and 7-methoxy-4-methylcoumarin (MMC)^17^ are fluorophores that are reported to emit fluorescence signals in polar media; unfortunately, they have limited applicability due to fluorescence quenching by protonation, difficulty of structural modification, and short excitation and emission wavelengths^18^. Other researchers have developed hydrogen-bonding-modulated fluorescent probes with strong fluorescence in protic solvents^18^,^19^, but their fluorescence is correlated with hydrogen-bonding donability rather than solvent polarity.

Lysosomes are sacs of hydrolytic enzymes within cells whose function is to break down unwanted macromolecules into basic molecules for cell recycling^20^,^21^. Lysosome dysfunctions, e.g., lysosome membrane permeabilization (LMP)^22^ and lysosome storage disorders (LSDs)^23^, are linked to cell death, several neurodegenerative disorders, cancer, cardiovascular diseases, and ageing-related diseases^24^,^25^,^26^. Depending on their functional state, lysosomes can vary considerably in structure, content, and internal environment^27^,^28^. Inherited LSDs are caused by a genetically determined deficiency of a lysosomal enzyme and are characterized by a significant augmentation of the lysosomal apparatus^23^ and an accumulation of polar lipids^29^ or water-soluble substrates^30^. The distinctive sign of LMP is the translocation of soluble lysosomal components from the lysosomal lumen to the cytosol^22^. These changes in lysosomal content are accompanied by alterations in the local polarity within cells. Thus, the determination of lysosomal polarity would be very helpful for the study of lysosomal physiology and pathology. Unfortunately, to the best of our knowledge, there are currently no polarity-sensitive fluorescent probes suitable for lysosomal applications.

Here, we report a rational design of a series of “off-on” polarity-sensitive fluorescent probes **BP** series that respond to highly polar media by emitting stronger fluorescence. Our approach relies on the polarity-dependent donor-excited photoinduced electron transfer (d-PET) mechanism that occurs between difluoroboron dippyomethene (BODIPY) fluorophore^31^,^32^,^33^,^34^,^35^,^36^ and the quaternary ammonium moiety. In addition, this study demonstrates that the novel polarity sensor **BP-2** has significant potential for detecting local hydrophilicity in cell lysosomes.

## Results

### Probe design and synthesis

To achieve “off-on” polarity sensing, we introduced positively charged quaternary ammonium to the *meso*-position of BODIPY fluorophore. In nonpolar media, the quaternary ammonium cation possesses low negative reduction potential (i.e., reduction was favored) and can be served as an efficient electron acceptor for the BODIPY fluorophore, thus allowing it to “switch off” BODIPY fluorescence *via* a d-PET process. As the solvent polarity increases, the reduction potential of the acceptor decreases (i.e., reduction was unfavored)^10^. Finally, the fluorescence can be revived in polar solvents because of the suppression of d-PET. In addition, the introduction of the quaternary ammonium moiety mediates the hydrophilic-lipophilic balance of the BODIPY fluorophore and imparts lysosome-targeting capability to the probes^37^.

**Figure 1:**
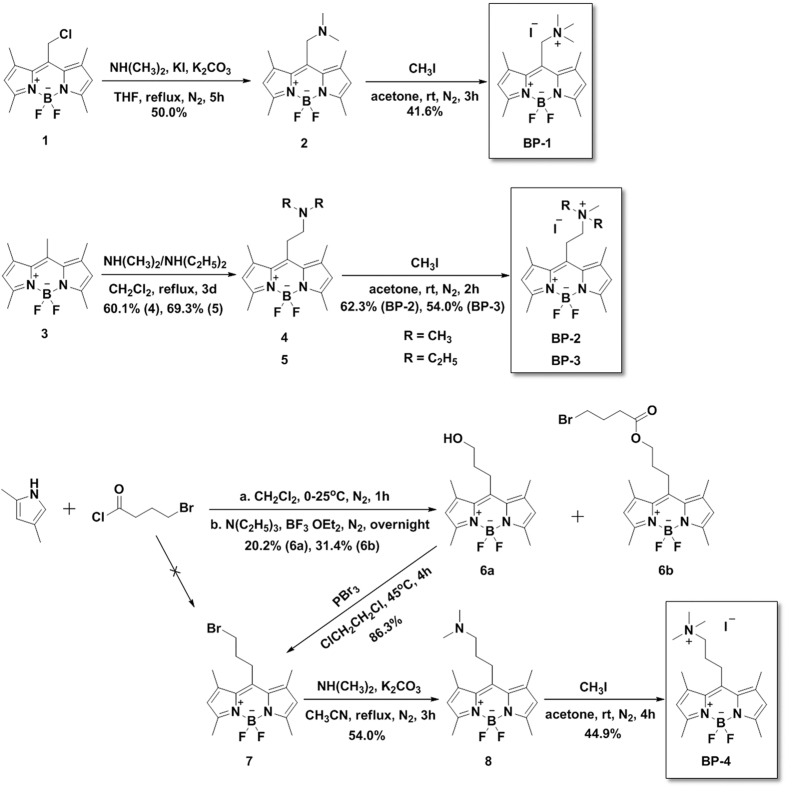
Synthesis of probes BP-1, BP-2, BP-3, and BP-4.

Figure 1 outlines the synthesis of these compounds. Briefly, amination of *meso*-chloromethyl-BODIPY **1**^38^ with dimethylamine furnished tertiary amine **2**. Subsequent quaternization gave probe **BP-1**. **BP-2** and **BP-3** were synthesized *via* an unusual Mannich-type reaction^39^ of *meso*-methyl-BODIPY **3**^40^ with dimethylamine or diethylamine in dichloromethane (DCM) and subsequent quaternization. In the synthesis of **BP-4**, condensation of 2,4-dimethylpyrrole with 4-bromobutanoyl and BF_3_•OEt_2_ was expected to generate *meso*-bromopropyl-BODIPY **7**. However, coupling of 4-bromobutanoyl and 2,4-dimethylpyrrole yielded dipyrromethene and water (see Supplementary Figure S1). Bromine was subsequently substituted by water under basic conditions (Et_3_N), followed by boron insertion with BF_3_•OEt_2_, which yielded *meso*-hydroxypropyl-BODIPY **6a**. Parts of **6a** were esterified with the remaining 4-bromobutanoyl to yield **6b**. Bromination of **6a** with PBr_3_ produced **7**, which was then aminated and quaternized to yield **BP-4**.

### Spectroscopic properties

**Figure 2:**
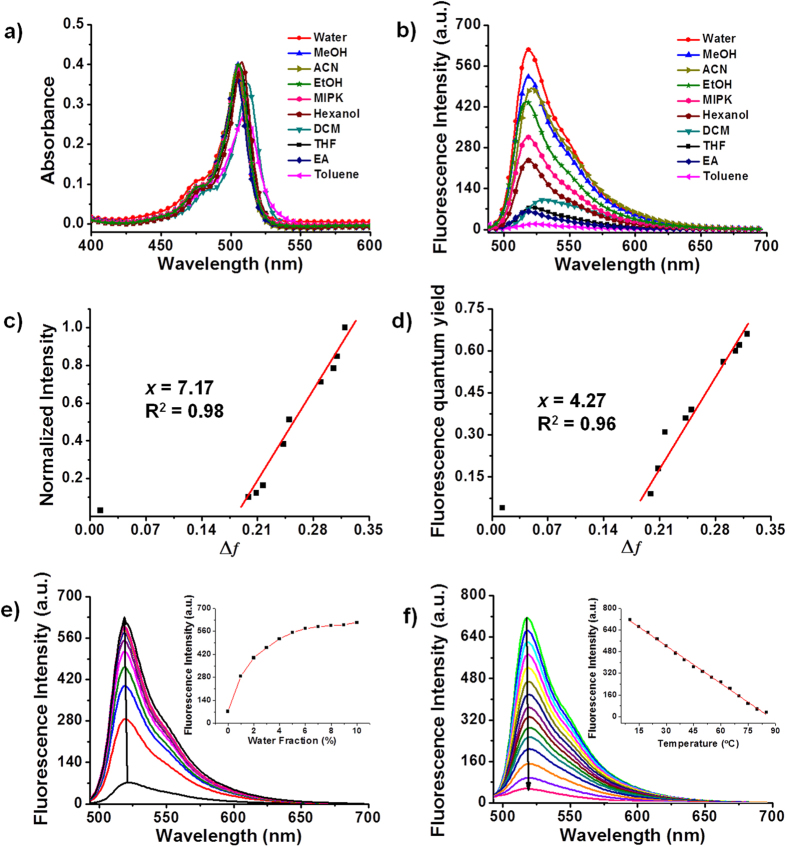
Absorption (**a**) and fluorescence (**b**) spectra of **BP-2** in different solvents (EA, ethyl acetate; THF, tetrahydrofuran; MIPK, methyl isopropyl ketone). Normalized fluorescence intensities (**c**) and fluorescence quantum yields (**d**) of **BP-2** as a function of the solvent orientational polarity parameter Δ*f*. (**e**) Fluorescence spectra and intensities (inset) of **BP-2** in THF with increasing amounts of water (0–10%). (**f**) Fluorescence spectra and intensities (inset) of **BP-2** at various temperatures. *λ*_ex_ = 480 nm.

We first investigated the spectroscopic properties of **BP-1** (see Supplementary Figure S2), **BP-2** (see Fig. 2 and Supplementary Table S1), **BP-3** (see Supplementary Figure S3), and **BP-4** (see Supplementary Figure S4) in several solvents that vary widely in polarity levels, as expressed by their orientation polarizability values (Δ*f*, see Supplementary Equation S1). The absorption spectra of the probes showed almost no appreciable changes in their maxima and intensities with varying Δ*f*. By contrast, the fluorescence spectra of the probes exhibited a prominent solvatofluoromism. All probes emitted weak fluorescence in apolar solvents but strong fluorescence in polar solvents. The emission intensities (*I*) and fluorescence quantum yields (Φ_*f*_) were strongly correlated with the solvent polarity. Compared to **BP-1** and **BP-4**, the two-carbon-linker probes **BP-2** and **BP-3** demonstrated higher linearity (*R*^2^ = 0.93–0.98) between *I* or Φ_*f*_  and Δ*f* in the range of 0.199–0.320 and lower background fluorescence (in toluene). To quantify the sensitivity of the probes, the slope (*x*) of the fitted line was determined. **BP-2** exhibited higher *x* values of 7.17 (*I*) and 4.27 (Φ_*f*_) than **BP-3**. These results indicated that **BP-2**, with a two-carbon linker and a trimethyl quaternary ammonium moiety, showed the best linear response and highest sensitivity to solvent polarity among the **BP** probes. Thus, **BP-2** was chosen for further investigation. The fluorescence lifetime (*τ*) of **BP-2** did not vary significantly in different solvents (*τ* ranged from 3.42 to 5.14 ns in all solvents, see Supplementary Table S1). The radiative decay rate constant (*k*_*r*_) and non-radiative decay rate constant (*k*_*nr*_) were calculated from *Φ*_*f*_ and *τ* using Supplementary Equation S2 and S3^41^. With increasing solvent polarity, the *k*_*r*_ value of **BP-2** increased while the *k*_*nr*_ value decreased, suggesting that non-radiative processes were suppressed and **BP-2** fluorescence was recovered in the polar solvents.

The specific interactions of **BP-2** with polar solvents were identified by examining its emission spectra in the binary solvents and temperature titration. Addition of a small amount of water to a solution of **BP-2** in tetrahydrofuran (THF) resulted in substantial spectral changes (Fig. 2e and see Supplementary Figure S5), which is a hallmark of a specific solvent effect^41^. The emission intensity of **BP-2** in aqueous solution decreased as the temperature increased (Fig. 2f), which is consistent with weaker fluorophore-solvent interactions at higher temperatures. Fortunately, BP-2 gave inconsiderable response to temperature within 35–40 °C which covers the range of general mammalian temperature (see Supplementary Figure S6a). In addition, the temperature-sensitive behavior of BP-2 was recyclable between 10 °C and 50 °C (see Supplementary Figure S6b). Given the quaternary ammonium in **BP-2** is positively charged, the solvent effect between **BP-2** and polar solvents is defined as the charge-dipole interactions^42^.

### Studies of the d-PET process in BP-2

The feasibility of electron transfer between a fluorophore and a quencher can be assessed by the Rehm-Weller equation (Equation 1)^43^,


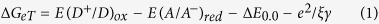


**Figure 3:**
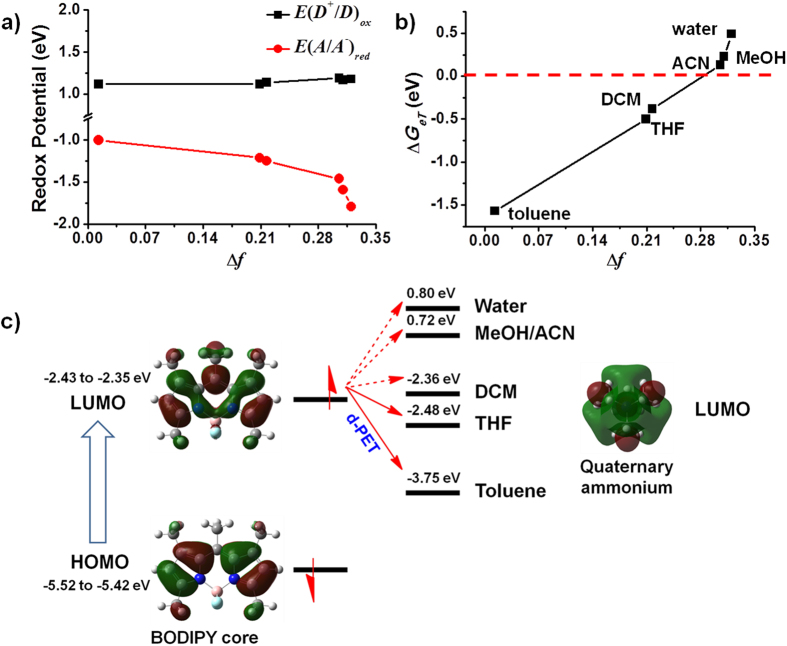
Electrochemical and theoretical studies of the d-PET process in BP-2. (**a**) Plots of *E*(*D*^+^/*D*)_*ox*_ and *E*(*A*/*A*^−^)_*red*_ against solvent polarity; (**b**) changes in Δ*G*_*eT*_ as a function of Δ*f*; (**c**) frontier orbital energy representation of the d-PET process in **BP-2** in different solvents.

Differential pulse voltammetry (DPV) was performed to determine the redox potentials of BODIPY and the quaternary ammonium cation in different solvents, including toluene, THF, DCM, methanol (MeOH), acetonitrile (ACN), and water. The oxidation potential of BODIPY maintained an almost constant value (from 1.12 eV in toluene to 1.18 eV in water) regardless of solvent polarity, while the reduction potential of the quaternary ammonium moiety significantly decreased (from −1.00 eV in toluene to −1.79 eV in water) as the solvent polarity increased (Fig. 3a). This result indicates that it is easier for the quaternary ammonium moiety to be reduced (i.e., to accept electrons) in less polar solvents. For the other parameters in the Rehm-Weller Equation, Δ*E*_0.0_ was essentially constant at 2.45 ± 0.01 eV, and *r* was estimated to be 5 Å by geometry optimization of **BP-2** using density functional theory (DFT) calculations. Taken together, the driving force (Δ*G*_*eT*_) in different solvents was calculated. As depicted in Fig. 3b, the Δ*G*_*eT*_ value increased with increasing solvent polarity (from −1.57 eV in toluene to 0.49 eV in water), suggesting that electron transfer from BODIPY to the quaternary ammonium cation (d-PET) in **BP-2** occurs more readily in solvents with lower polarity.

Frontier orbital energy diagrams were also constructed according to the DFT method to investigate the fluorescence signal output of **BP-2** in solvents with different polarities (Fig. 3c). The HOMO (−5.52 to −5.42 eV) and LUMO (−2.43 to −2.35 eV) levels of BODIPY remained almost unchanged in all solvents. For the quaternary ammonium cation, the LUMOs varied significantly with solvent polarity. In toluene and THF, d-PET was favored because the LUMOs of the cation (−3.75 eV for toluene and −2.48 eV for THF) were located between the HOMO and LUMO of BODIPY. However, in polar solvents, the LUMO of the cation rose far above that of BODIPY (0.72 eV for MeOH and ACN, 0.80 eV for water), thus inhibiting the d-PET process and restoring the fluorescence. The HOMO of the cation was not taken into account due to its much lower location compared to that of BODIPY (see Supplementary Figure S7). Taken above, we conclude that the “off-on” response of **BP-2** fluorescence from apolar to polar solvents is primarily ascribed to the polarity-modulated d-PET process.

One may concern that the varied solubilities of **BP-2** in different solvents may involve in its solvent-dependent fluorescence. As depicted in Supplementary Figure S8, the absorbance of **BP-2** increased linearly with **BP-2** concentration (*R*^2^ = 0.99) in the range of 1–10 μM in all solvents. No aggregation was detected by dynamic light scattering (DLS) at a concentration of 10 μM (data not shown), even in toluene and ACN, which are usually used to precipitate quaternary ammonium salts.

### Evaluation of BP-2 selectivity

A set of assays was performed to evaluate **BP-2** selectivity for solvent polarity (see Supplementary Figure S9). First, we investigated the sensitivity of **BP-2** to solution viscosity in a binary solution of glycerol and ethanol, solvents with similar polarities (Δ*f* = 0.274 and 0.290 for glycerol and ethanol, respectively). When the ratio of glycerol to ethanol was increased, only a slight increase in **BP-2** fluorescence emission (0.3-fold) was observed, which may be attributed to inhibited vibration and rotation of the methyl groups on the BODIPY skeleton. A pH titration study revealed that **BP-2** fluorescence maintained a constant maximum value in buffered aqueous solutions over a wide range of pH values (pH 4–10). Additionally, we evaluated the fluorescence response of **BP-2** to a variety of biologically relevant species, including ions, amino acids, reactive oxygen species (ROS), nucleic acids, and proteins. The presence of these species did not significantly alter the fluorescence spectrum of **BP-2**. Altogether, these results indicate that **BP-2** has potential as a specific polarity sensor in complicated biological environments.

### Subcellular distribution of BP-2

**Figure 4:**
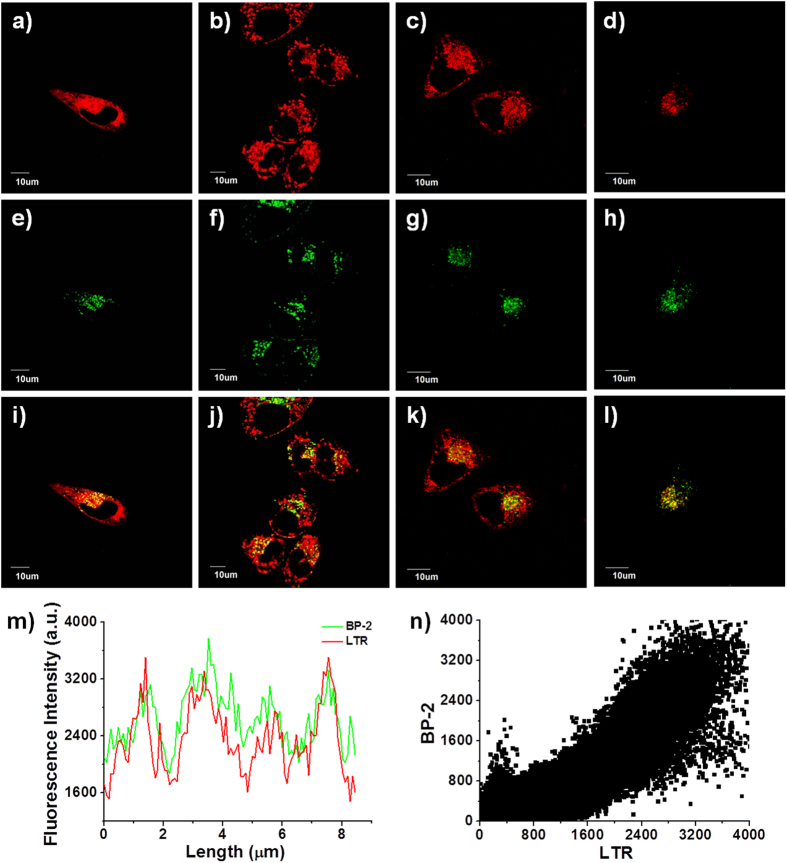
Confocal fluorescence images of MCF-7 cells co-labeled with (**e**–**h**) **BP-2** (5 μM) and commercial organelle markers: (**a**) ER-Tracker Red (1 μM); (**b**) MitoTracker Deep Red (500 nM); (**c**) BODIPY TR ceramide (1 μM); (**d**) LysoTracker Red (100 nM). (**i**–**l**) Merged images of (**a**,**e**), (**b**,**f**), (**c**,**g**), (**d**,**h**), respectively. (**m**) Intensity profile of the region of interest (ROI) across the cell in (**l**). (**n**) Correlation plot of **BP-2** and LysoTracker Red fluorescence intensities.

Next, we turned our attention to evaluate **BP-2** in live-cell imaging assays. After MCF-7 cells were incubated with 5 μM **BP-2** for 20 min at 37 °C, **BP-2** penetrated through the cell membrane and stained the cells. Punctate fluorescence was observed near perinuclear regions (see Supplementary Figure S10) in the green wavelength range (490 nm to 550 nm). The fluorescence spectra extracted from cell images was nearly identical to that of **BP-2** measured in water. Subsequently, the subcellular distribution of **BP-2** was determined by co-staining experiments (Fig. 4) with commercially available organelle markers. **BP-2** fluorescence overlapped significantly with that of LysoTracker Red (yellow areas in merged images), whereas little overlap was observed with other markers (Fig. 4i–l). The changes in fluorescence intensities for **BP-2** and LysoTracker Red were almost synchronized (Fig. 4m). From the correlation plots, a high Pearson’s coefficient (*R*_r_ = 0.95) was obtained for **BP-2** with LysoTracker Red co-staining (Fig. 4n). These observations suggest that **BP-2** can specifically localize to lysosomes in living cells.

### Reporting changes in local hydrophilicity within lysosomes

**Figure 5:**
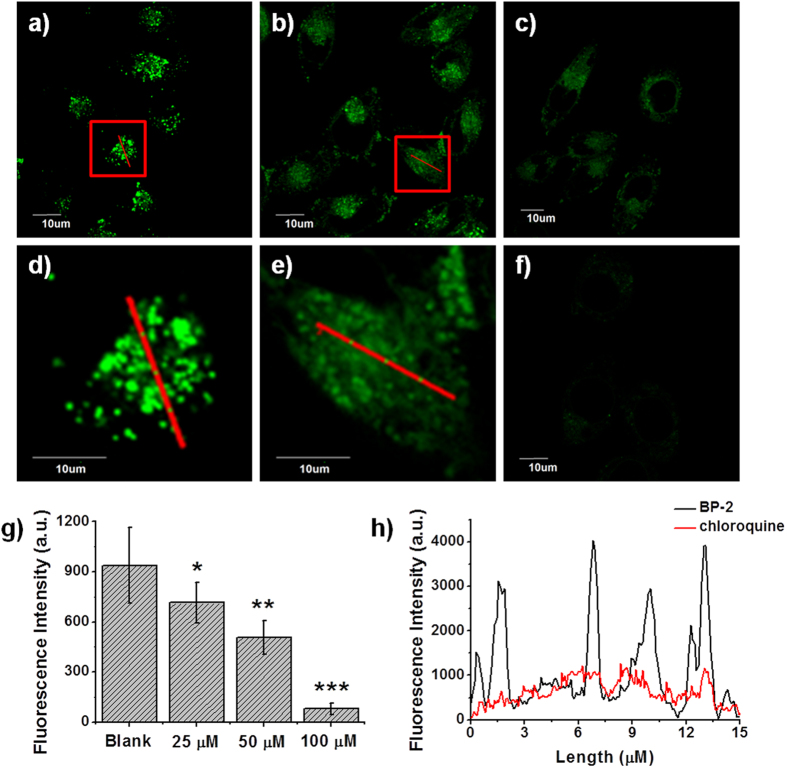
Confocal fluorescence images of MCF-7 cells stained with 5 μM **BP-2** and stimulated with different concentrations of chloroquine: (**a**) blank; (**b**) 25 μM; (**c**) 50 μM; (**f**) 100 μM. (**d**,**e**) Enlarged images of representative cells (red squares) in (**a**,**b**). (**g**) Statistical analyses performed with the two-sample *t*-test (*n* = 10 fields of cells). *P < 0.05, **P < 0.01, ***P < 0.001; error bars are ± sem. (**h**) Intensity profile of the ROIs across the cell in (**d**,**e**).

We tested the utility of **BP-2** in the visualization of chloroquine-induced LMP^44^. **BP-2**-prestained MCF-7 cells were treated with different concentrations of chloroquine (25, 50 and 100 μM) and the fluorescence images were recorded immediately. The lysosome-characteristic punctate fluorescence of **BP-2** disappeared upon stimulation by chloroquine (25 μM) and was replaced by diffuse staining throughout the cytoplasm (Fig. 5b,e). The intensity profile (Fig. 5h) clearly displayed the substantial difference in the distribution of **BP-2** fluorescence between before and after the addition of chloroquine in living cells: accumulating in specific regions (**BP-2** only); almost uniformly distribution (in the presence of chloroquine). To quantify fluorescence intensity, ten regions of interest were selected for each image, and the average fluorescence over these areas was calculated. **BP-2** fluorescence gradually decreased as the concentration of chloroquine increased (Fig. 5g). After treatment with 100 μM chloroquine, the relative emission intensity of **BP-2** sharply decreased from 939 ± 226 to 79 ± 34. Note that the fluorescence of **BP-2** was independent of chloroquine in the phosphate-buffered saline (PBS) buffer (see Supplementary Figure S11), which indicates that the decrease in local hydrophilicity was responsible for the observed variations in **BP-2** fluorescence.

**Figure 6:**
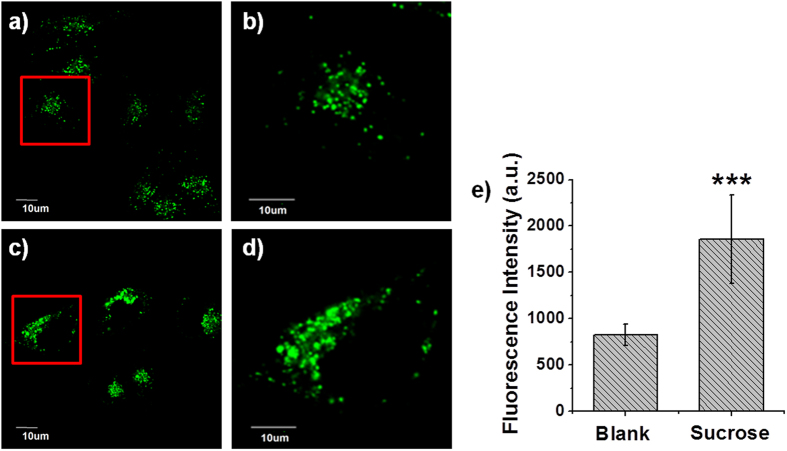
Confocal fluorescence images of MCF-7 cells stained with 5 μM **BP-2** before (**a**,**b**) and after (**c**,**d**) the addition of 80 mM sucrose. (**b**,**d**) Enlarged images of representative cells (red squares) in (**a**,**c**). (**e**) Statistical analysis performed with the two-sample *t*-test (*n* = 10 fields of cells). ***P < 0.001; error bars are ± sem.

Finally, we used **BP-2** to characterize the mimic LSD induced by high sucrose. MCF-7 cells pre-labelled with **BP-2** were loaded with 80 mM sucrose and then imaged with confocal fluorescence microscope immediately. Lysosomes swelled in the presence of sucrose (Fig. 6c,d), accompanied by a remarkable increase in **BP-2** fluorescence. The relative fluorescence intensity of **BP-2** increased from 825 ± 117 to 1857 ± 475 after sucrose treatment (Fig. 6e). No detectable change in fluorescence was observed when 80 mM sucrose was added to the buffer solution of **BP-2** (see Supplementary Figure S11), demonstrating that the fluorescence enhancement was due to the increase in lysosomal local hydrophilicity. These results suggest that massive sucrose induces a more polar environment within lysosomes.

## Discussion

Polarity-sensitive fluorescent probes offer a unique opportunity for non-invasive, *in situ* determination of microenvironmental polarity in biological systems^3^. Much effort has been devoted to the development of polarity-sensitive “on-off” probes to study hydrophobic interactions and structures^4^,^5^,^6^,^7^,^8^,^9^. However, there is a lack of “off-on” polarity-sensing probes for studying and imaging biological processes that involve in an increase in local hydrophilicity. In this work, we designed a series of polarity-sensitive fluorescent probes whose “off-on” mechanism operates based on the sensitivity of the redox potential of quaternary ammonium to solvent polarity. In apolar solvents, the quaternary ammonium is capable of quenching the fluorescence of BODIPY through a d-PET process due to its large electron deficiency and high reduction potential. As the environmental polarity increases, the charge-dipole interaction between the quaternary ammonium and polar solvent molecules decreases the reduction potential of quaternary ammonium. An increase in polarity reduces the ability of quaternary ammonium to accept electrons, thus blocking the d-PET process and recovering BODIPY fluorescence. We examined the effect of linker length and size of quaternary ammonium on the ability of the probes to sense changes in polarity. We found that a two-carbon linker is the optimal spacer for the electron transfer from BODIPY to the quaternary ammonium, and that the small size of trimethyl quaternary ammonium facilitates its specific interaction with polar molecules.

Lysosomes serve as the dispatch center for cell recycling^21^ and have significant functions in the pathogenesis of various disorders and diseases^24^,^25^,^26^. The morphology and contents of lysosomes are variable depending on their functional state^27^,^28^. Alterations in the internal environment of lysosomes under pathological conditions are reflected by changes in lysosomal polarity. Therefore, lysosomal polarity-sensing probes may be useful tools that can improve our understanding of lysosome-related activities and disorders. Although several existing polarity-sensitive probes show specific subcellular distributions^9^,^10^,^11^,^12^,^14^, none of these probes are designed to target lysosomes. In this study, **BP-2** has been proven to be the first polarity-sensitive probe that specifically localized to lysosomes in living cells. Moreover, we utilized **BP-2** in confocal fluorescence imaging to report the changes in local hydrophilicity within lysosomes under chloroquine-induced LMP (decrease) and mimic LSD (increase).

In summary, we have developed a series of “off-on” polarity-sensitive fluorescent probes (**BP-1**, **BP-2**, **BP-3** and **BP-4**) that respond to highly polar media by emitting a higher fluorescence signal *via* a solvent-dependent d-PET mechanism. Among these probes, **BP-2**, with a two-carbon linker and a trimethyl quaternary ammonium moiety, exhibited the best polarity-sensing properties: linear response, high sensitivity and specificity. Significantly, using **BP-2**, we present the first fluorescence images that reflect local hydrophilicity within lysosomes under conditions of lysosomal dysfunction. Our work opens up a new arena for the design of “off-on” polarity-sensitive fluorescent probes and provides a novel probe (**BP-2**) that shows significant potential as a chemical tool for the study of lysosomal biology and the diagnosis of lysosome-related disorders.

## Methods

### General information

General chemicals were of analytical grade without further purification. All the solvents employed were of spectrometric grade. Solutions of ions were prepared from FeCl_3_ · 6H_2_O, CrCl_3_, MnCl_2_ · 5H_2_O, CoCl_2_ · 6H_2_O, NiCl_2_ · 6H_2_O, CuCl_2_ · 2H_2_O, KCl, NaCl, MgCl_2_ · 6H_2_O, CaCl_2_, Na_2_CO_3_, Na_2_SO_3_, Na_3_PO_4_, NaF, KBr, NaNO_3_, NaAc, NaClO_4_ dissolved in distilled water. Solutions or generation of ROS were prepared according to our previous report^45^. Amino acids, proteins, and nucleic acids were dissolved in distilled water to prepare stock solutions. Stock solutions (5 mM) of **BP** series were prepared in dimethyl sulphoxide (DMSO) and stored in a refrigerator for use. Organelle markers were purchased from Invitrogen (USA). Sucrose was obtained from Energy Chemical (China). Chloroquine was from Sigma-Aldrich (USA). ^1^H-NMR and ^13^C-NMR spectra were recorded on a VARIAN INOVA-400 spectrometer with chemical shifts (*δ*) reported as ppm (in CDCl_3_ or d^6^-DMSO, TMS as the internal standard). Mass spectrometry data were obtained with an HP1100LC/MSD mass spectrometer and an LC/Q-TOF MS spectrometer. Absorption spectra were measured on a Lambda 35 UV/VIS spectrophotometer (Perkin Elmer). Fluorescence measurements were performed on a VARIAN CARY Eclipse fluorescence spectrophotometer (Serial No. FL0812-M018). Excitation and emission slit widths were modified to adjust the fluorescence intensity to a suitable range. The fluorescence lifetimes were attained from a Horiba Jobin Yvon Fluoromax-4p. Slight pH variations in the solutions were achieved by adding the minimum volumes of NaOH or HCl (1 M). All pH measurements were made with a Model PHS-3C meter. For the viscosity sensitivity experiment^34^, **BP-2** was added to the solvent mixtures of ethanol and glycerol in different proportions. The final solutions were sonicated for 5 min to eliminate air bubbles. After standing for 1 h at r. t., the fluorescence measurements were performed.

### Live-cell imaging experiments

MCF-7 cells were cultured in Dulbecco’s modified Eagle’s medium (Invitrogen) supplemented with 10% fetal bovine serum (Invitrogen). Cells were seeded in 24-well flat-bottomed plates and incubated for 24 h at 37 °C under 5% CO_2_. **BP-2** (5 μM) was added (the concentration of DMSO was maintained to be less than 0.2%) and cells were further incubated for 20 min, followed by washing thrice with phosphate-buffered saline (PBS). The fluorescence imaging was performed with OLYMPUS FV-1000 inverted fluorescence microscope with 100× objective lens. Under the confocal fluorescence microscope, **BP-2** was excited at 488 nm and emission was collected at 490–550 nm. Co-localization experiments were conducted by co-staining the cells with combinations of **BP-2** and ER-Tracker Red (1 μM, *λ*_ex_ = 559 nm, *λ*_em_ = 570–650 nm)/MitoTracker Deep Red (500 nM, *λ*_ex_ = 635 nm, *λ*_em_ = 655–755 nm)/BODIPY TR ceramide (1 μM, *λ*_ex_ = 559 nm, *λ*_em_ = 570–650 nm)/LysoTracker Red (100 nM, *λ*_ex_ = 559 nm, *λ*_em_ = 570–650 nm) for 20 min. For the LMP experiment, MCF-7 cells internalized with **BP-2** (5 μM, 20 min) were treated with chloroquine (25, 50 and 100 μM). For the sucrose-stimulation experiment, MCF-7 cells were incubated with **BP-2** (5 μM, 20 min) and then sucrose (80 mM) was added.

## Additional Information

**How to cite this article**: Zhu, H. *et al*. d-PET-controlled “off-on” Polarity-sensitive Probes for Reporting Local Hydrophilicity within Lysosomes. *Sci. Rep.* **6**, 35627; doi: 10.1038/srep35627 (2016).

## Supplementary Material

Supplementary Information
