# Supplementary material for: d-PET-controlled “off-on” Polarity-sensitive Probes for Reporting Local Hydrophilicity within Lysosomes
Source: Sci Rep. 2016 Oct 21;6:35627. doi: 10.1038/srep35627 (PMC5073283; doi:10.1038/srep35627)
Supplement: Supplementary Information [file srep35627-s1.pdf]

d-PET-controlled “off-on” Polarity-sensitive Probes for Reporting Local Hydrophilicity within Lysosomes

Hao Zhu, Jiangli Fan,* Huiying Mu, Tao Zhu, Zhen Zhang, Jianjun Du, and Xiaojun Peng

State Key Laboratory of Fine Chemicals, Dalian University of Technology, 2 Linggong Road, Dalian, 116024, China

Email: fanjl@dlut.edu.cn

Content

Supplementary Figure S1. .... S3

Supplementary Equation S1........ S3

Supplementary Equation S2........ S3

Supplementary Equation S3........ S3

Supplementary Table S1. .... S4

Supplementary Figure S2. .... S5

Supplementary Figure S3. .... S6

Supplementary Figure S4. .... S7

Supplementary Figure S5. .... S7

Supplementary Figure S6. .... S8

Supplementary Figure S7. .... S8

Supplementary Figure S8. .... S9

Supplementary Figure S9. ....S10

Supplementary Figure S10. ....S11

Supplementary Figure S11. ....S11

Electrochemical measurements. S11

Theoretical calculation. S12

Determination of quantum yields. S12

Probe synthesis and characterization. S12

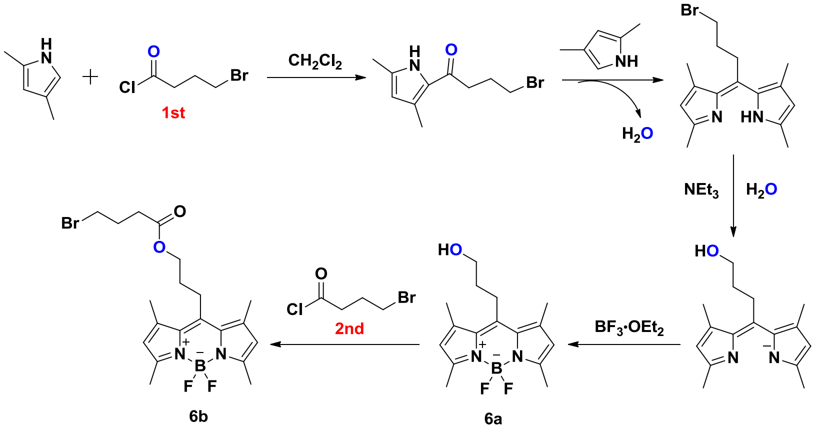

Supplementary Figure S1. Proposed synthesis mechanism of **6a** and **6b**

Calculation of the Lippert-Mataga solvent parameter Δ*f*

$$\Delta f = \frac{\varepsilon - 1}{2\varepsilon + 1} - \frac{n^2 - 1}{2n^2 + 1} \qquad \text{(Supplementary Equation S1)}$$

where ε is the dielectric constant and n is the refractive index of the solvent.

Calculation of radiative decay rate constant (*k*$_r$) and non-radiative decay rate constant (*k*$_{nr}$)

$$k_r = \frac{\Phi_f}{\tau} \qquad \text{(Supplementary Equation 2)}$$

$$k_{nr} = \frac{1 - \Phi_f}{\tau} \qquad \text{(Supplementary Equation 3)}$$

where Φ_f is the fluorescence quantum yield and τ is the fluorescence lifetime.

Supplementary Table S1. Photophysical data of **BP-2** in different solvents

Solvents	λ_{abs} (nm)	λ_{em} (nm)	ε [a]	Φ_f [b]	Δf [c]	τ (ns) [d]	k_r (10^7 s^{-1}) [e]	k_{nr} (10^7 s^{-1}) [f]
Toluene	510	523	5.53	0.04	0.013	3.42	1.17	28.07
EA	504	518	7.26	0.09	0.199	3.45	2.61	26.38
THF	507	522	7.86	0.18	0.209	4.12	4.37	19.90
DCM	512	530	7.05	0.31	0.217	5.14	6.03	13.42
Hexanol	508	521	8.13	0.36	0.243	4.91	7.33	13.03
MIPK	507	519	3.17	0.39	0.250	5.14	7.59	11.87
EtOH	506	520	8.02	0.56	0.290	4.64	12.07	9.48
ACN	505	519	7.79	0.60	0.306	4.96	12.10	8.06
MeOH	505	519	8.00	0.62	0.310	4.44	13.96	8.56
Water	505	521	7.29	0.66	0.320	4.70	14.04	7.23

[a] Molar extinction coefficient, $\times 10^4$ mol^{-1} cm^{-1} L. [b] rhodamine B was used as a standard reference with a fluorescence quantum yield of 0.49 in ethanol. [c] Solvent polarity scale of orientation polarizability. [d] Fluorescence lifetime. [e] Radiative decay rate constant. [f] Non-radiative decay rate constant.

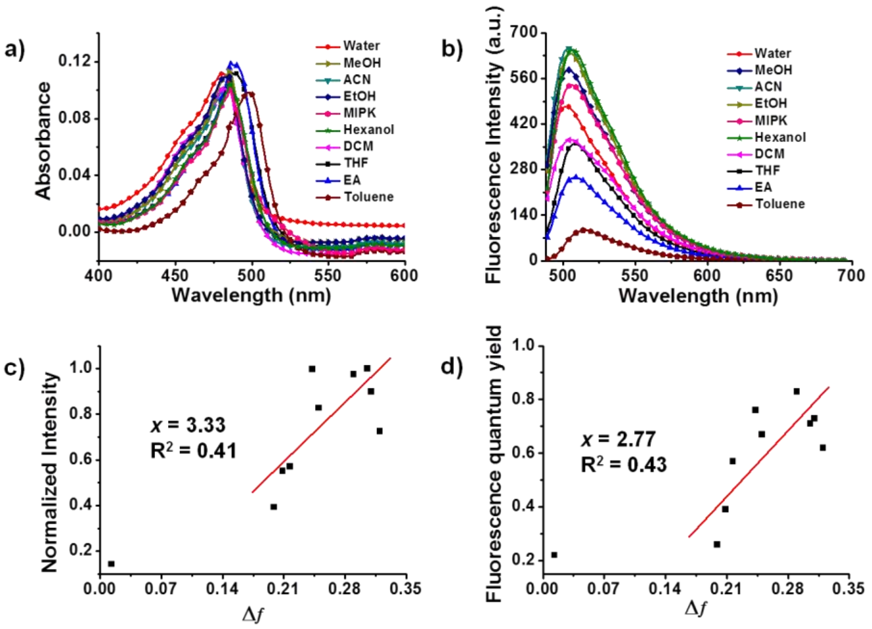

Supplementary Figure S2. Absorption (a) and fluorescence (b) spectra of **BP-1** in different solvents. Normalized fluorescence intensities (c) and fluorescence quantum yields (d) of **BP-1** as a function of the solvent orientational polarity parameter Δf. λ_{ex} = 480 nm.

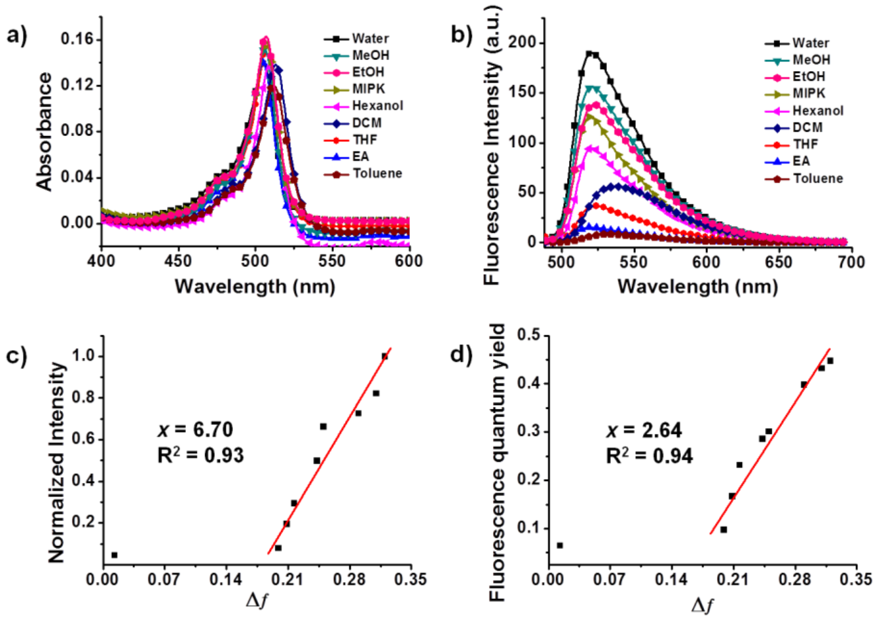

Supplementary Figure S3. Absorption (a) and fluorescence (b) spectra of **BP-3** in different solvents. Normalized fluorescence intensities (c) and fluorescence quantum yields (d) of **BP-3** as a function of the solvent orientational polarity parameter Δf. λ_{ex} = 480 nm.

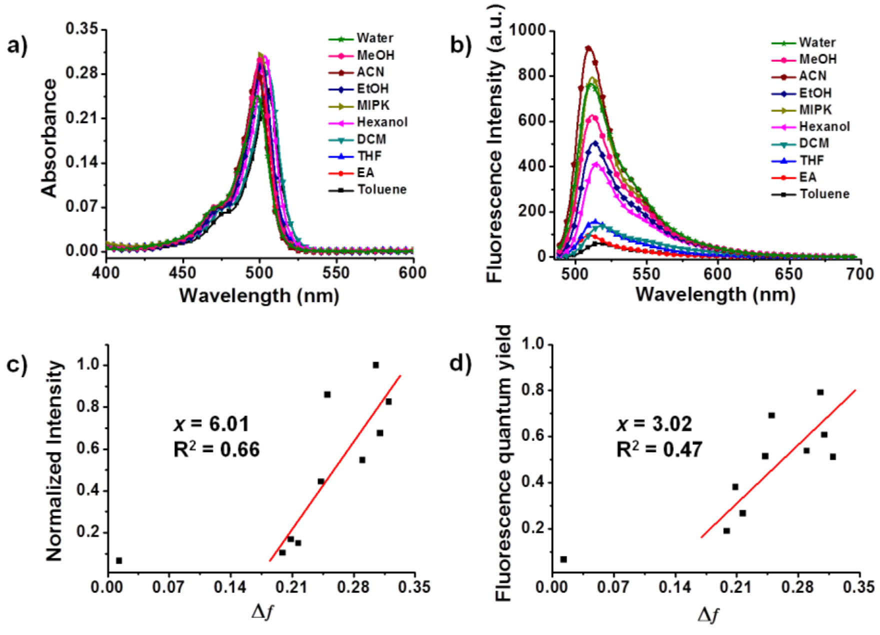

Supplementary Figure S4. Absorption (a) and fluorescence (b) spectra of **BP-4** in different solvents. Normalized fluorescence intensities (c) and fluorescence quantum yields (d) of **BP-4** as a function of the solvent orientational polarity parameter Δf. λ_{ex} = 480 nm.

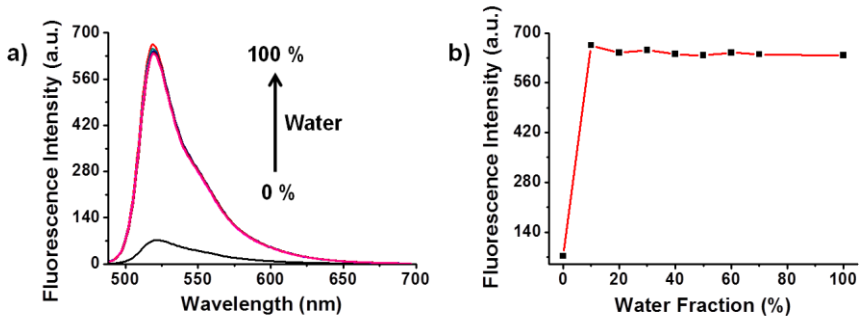

Supplementary Figure S5. Fluorescence spectra (a) and intensities (b) of **BP-2** in THF with increasing amounts of water (0-100%). λ_{ex} = 480 nm.

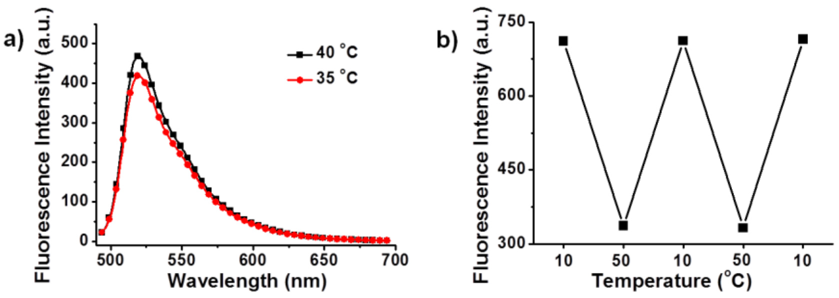

Supplementary Figure S6. a) Fluorescence spectra of **BP-2** at 35 ^{o}C (red) and 40 ^{o}C (black). λ_{ex} = 480 nm. b) Recyclable temperature-sensitive behavior of **BP-2** between 10 ^{o}C and 50 ^{o}C.

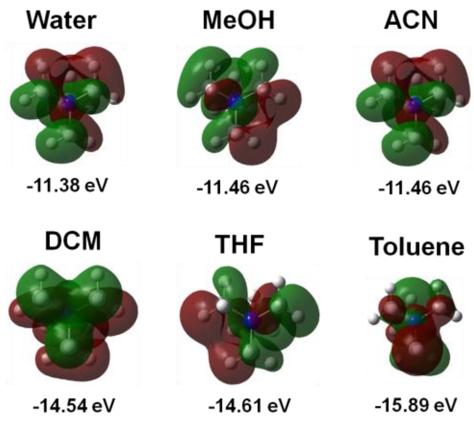

Supplementary Figure S7. HOMO levels of the quaternary ammonium moiety in different solvents.

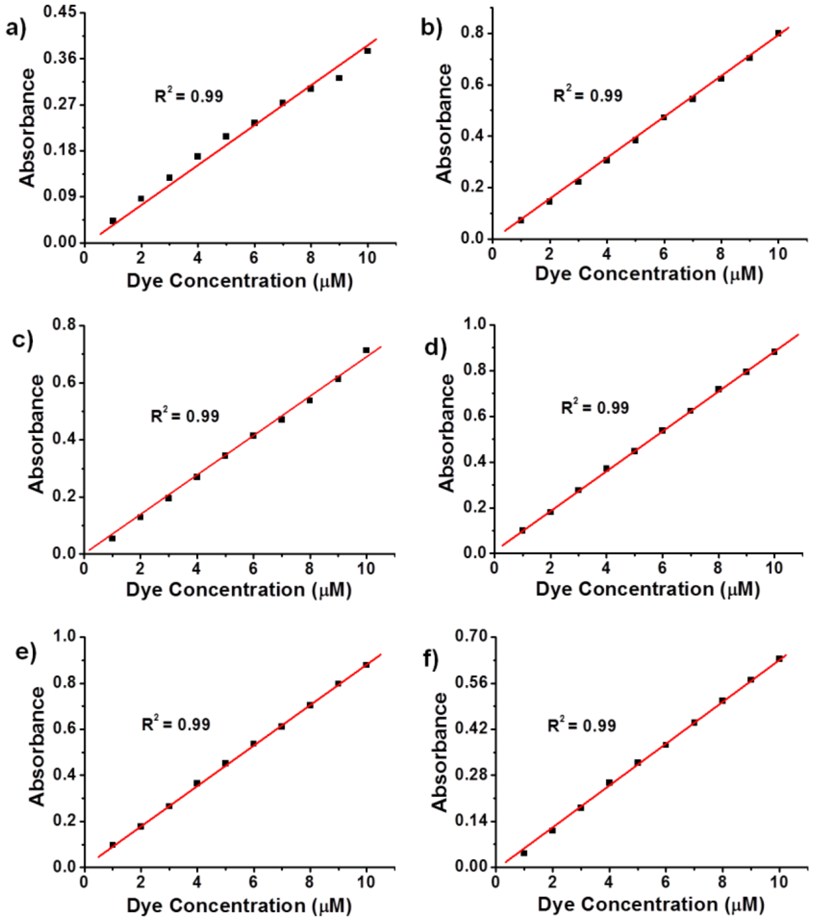

Supplementary Figure S8. Absorbance of **BP-2** at maximum wavelength with increasing dye concentration in toluene (a), THF (b), DCM (c), ACN (d), MeOH (e), and water (f).

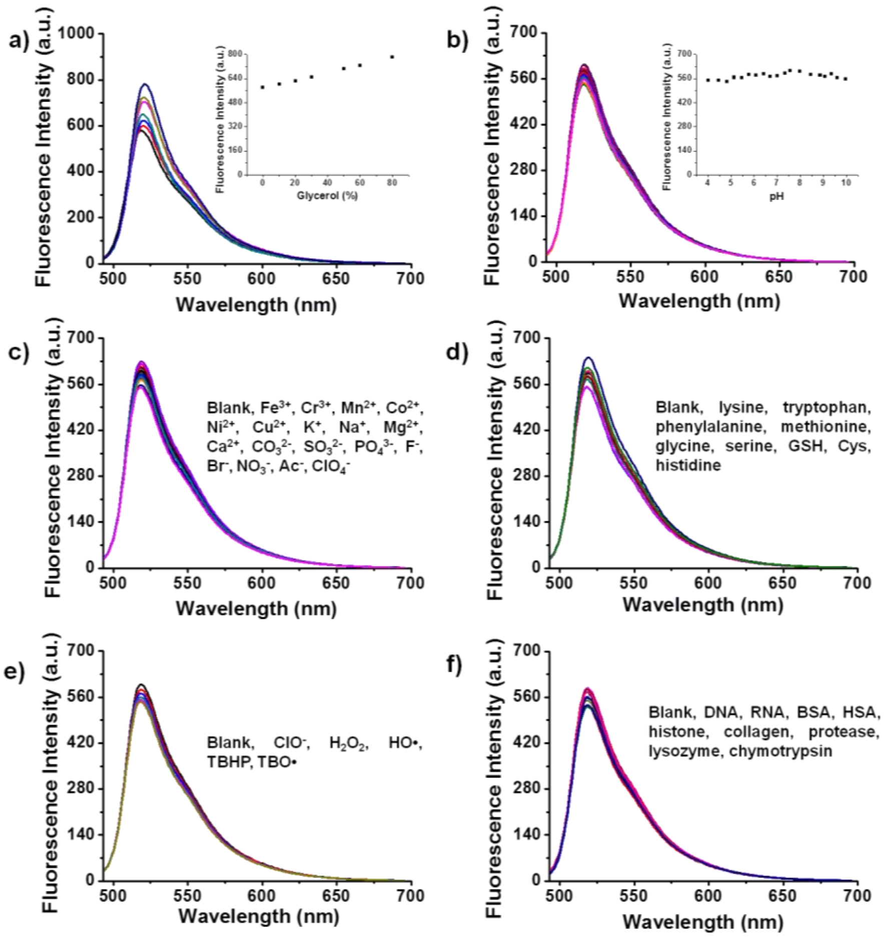

Supplementary Figure S9. Selectivity test of **BP-2**. a) Fluorescence spectra of **BP-2** with the variation of solution viscosity (ethanol/glycerol system). b) Effect of pH (range from 4 to 10) on the fluorescence properties of **BP-2**. c-f) Fluorescence spectra of **BP-2** in the presence of a variety of biologically relevant species: c) ions (1 mM for Ca^{2+}, Mg^{2+}, K^{+}, and Na^{+}, 50 μM for the others); d) amino acids (100 μM); e) ROS (10 μM); f) nucleic acids (20 μg/mL) and proteins (100 mg/L). Conditions: 0.02 M PBS buffer solution, pH = 7.4, λ_{ex} = 480 nm.

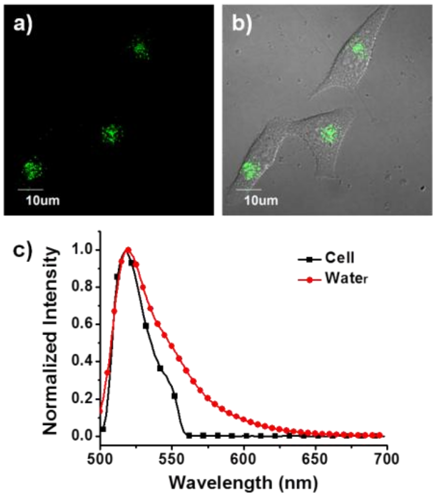

Supplementary Figure S10. a, b) Fluorescence images of **BP-2**-labelled MCF-7 cells. The excitation wavelength was 488 nm and the fluorescence was collected at 490-550 nm. c) Normalized fluorescence spectra of **BP-2** in water (red) and living cells (black).

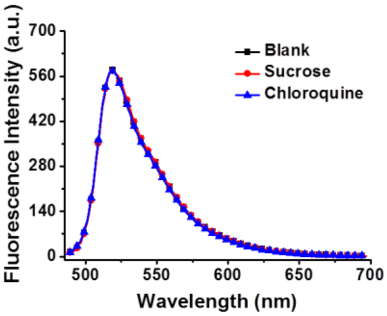

Supplementary Figure S11. Fluorescence spectra of **BP-2** (5 μM) in the prescence of sucrose (80 mM) or chloroquine (100 μM) in 0.02 M PBS buffer solution (pH = 7.4). λ_{ex} = 480 nm.

Electrochemical measurements

The differential pulse voltammetry (DPV) measurements were taken using a CHI660D electrochemical workstation and carried out in 0.1 M of $(n\text{-}Bu)_4NPF_6$/ACN at a scan rate of 100 mV/s. The working electrode was a glassy carbon with a

diameter of 0.3 cm. A platinum wire coil was used as the counter electrode. All potentials are referenced to an Ag/Ag^+ electrode in ACN. The tested compound concentration was 5×10^{-4} M.

Theoretical calculation

All the quantum-chemical calculations were done with the Gaussian 09 suite.[1] The parameter referred to the previous work.[2,3] The geometry optimizations of the dyes were performed using density functional theory (DFT) with Becke's three-parameter hybrid exchange function with Lee-Yang-Parr gradient-corrected correlation functional (B3-LYP functional) and 6-31G** basis set.[4] No constraints to bonds/angles/dihedral angles were applied in the calculations and all atoms were free to optimize.

Determination of quantum yields

The fluorescence quantum yields of **BP-1**, **BP-2**, **BP-3**, and **BP-4** in different solvents were determined according to the method below,[5]

$$\varphi_u = \frac{(\varphi_s)(FA_u)(A_s)(\lambda_{exs})(\eta_u^2)}{(FA_s)(A_u)(\lambda_{exu})(\eta_s^2)}$$

where φ is fluorescence quantum yield; *FA* is integrated area under the corrected emission spectra; *A* is the absorbance at the excitation wavelength; λ_{ex} is the excitation wavelength; η is the refractive index of the solution; the subscripts *u* and *s* refer to the unknown and the standard, respectively. We chose rhodamine B as standard, which has a fluorescence quantum yield of 0.49 in ethanol.[6]

Probe synthesis and characterization

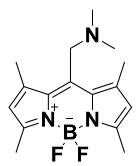

2

Compound 1[7] (90.0 mg, 0.3 mmol), dimethylamine (0.15 mL, 2 M in THF, 0.3 mmol), potassium iodide (7.5 mg, 0.045 mmol), and potassium carbonate (63.0 mg, 0.45 mmol) were added to dry THF (10 mL). The mixture was refluxed for 5 h under N_2 atmosphere. After filtration, THF was removed under reduced pressure by rotary evaporation. The crude product was purified by column chromatography (silica, CH_2Cl_2/MeOH = 200/7, v/v) to obtain compound 2 (46.0 mg, 50.0 %). ^{1}H NMR (400 MHz, $CDCl_3$), δ: 6.96 (s, 1H, pyrrole-H), 6.21 (s, 1H, pyrrole-H), 4.35 (s, 2H, CH_2), 2.74 (s, 6H, CH_3), 2.66 (s, 3H, CH_3), 2.54 (s, 3H, CH_3), 2.47 (s, 6H, CH_3). ^{13}C NMR (100 MHz, $CDCl_3$), δ: 157.6, 144.2, 143.3, 139.7, 133.5, 131.6, 129.5, 122.9, 121.3, 54.5, 44.1, 17.6, 17.4, 16.7, 14.7. TOF MS: m/z calcd for $C_{16}H_{23}BF_2N_3^+$ $[M+H]^+$ 306.1953, found: 306.1951.

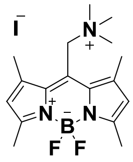

BP-1

Methyl iodide (0.9 mmol, 0.056 mL) was added dropwise to the solution of compound **2** (0.18 mmol, 55 mg) in acetone (3 mL). Then the mixture was stirred at 25 $^{\circ}$C under N_2 atmosphere for 3 h, and the solid product **BP-1** (33.5 mg ,41.6%) was obtained by filtration and being washed with acetone. ^{1}H NMR (400 MHz, d^6-DMSO), δ: 6.62 (s, 1H, pyrrole-H), 6.46 (s, 1H, pyrrole-H), 4.63 (s, 2H, CH_2), 3.07 (s, 9H, CH_3), 2.45 (s, 3H, CH_3), 2.45 (s, 3H, CH_3), 2.44 (s, 6H, CH_3). ^{13}C NMR (100 MHz, d^6-DMSO), δ: 155.2, 144.2, 143.7, 137.9, 134.1, 131.4, 130.1, 122.6, 118.9, 61.2, 53.6, 17.4, 16.8, 14.6. TOF MS: m/z calcd for $C_{17}H_{25}BF_2N_3^+$ $[M]^+$ 320.2104, found: 320.2116.

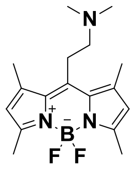

4

Dimethylamine (3.14 mL, 2 M in THF, 6.28 mmol) was added to a solution of compound **3**[8] (82 mg, 0.314 mmol) in dry CH_2Cl_2 (3.0 mL) in a reaction vial. After the reaction mixture was refluxed for 3 h, the resulting products were diluted with CH_2Cl_2 and washed with sat. NH_4Cl solution and brine. The organic layer was dried over $MgSO_4$ and concentrated under reduced pressure. The residue was purified by column chromatography (silica, CH_2Cl_2/MeOH = 200/4, v/v) to obtain compound **4** (60.0 mg, 60.1 %). ^{1}H NMR (400 MHz, $CDCl_3$), δ: 6.06 (s, 2H, pyrrole-H), 3.20 (t, J = 8 Hz, 2H, CH_2), 2.56 (t, J = 8 Hz, 2H, CH_2), 2.51 (s, 6H, CH_3), 2.47 (s, 6H, CH_3), 2.35 (s, 6H, CH_3). ^{13}C NMR (100 MHz, $CDCl_3$), δ: 154.2, 140.4, 137.4, 131.7, 121.8, 60.6, 45.4, 27.1, 16.6, 14.5. TOF MS: m/z calcd for $C_{17}H_{25}BF_2N_3^+$ $[M+H]^+$ 320.2104, found: 320.2115.

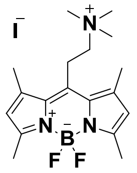

BP-2

Compound **4** (0.19 mmol, 60 mg) was dissolved in acetone (2 mL). Methyl iodide (0.94 mmol, 0.06 mL) was added dropwise to the above solution, and the mixture was stirred at 25 ^oC under N_2 atmosphere for 2 h. The solid product **BP-2** (54 mg, 62.3 %) was obtained by filtration and being washed with acetone. ^{1}H NMR (400 MHz, d^6-DMSO), δ: 6.33 (s, 2H, pyrrole-H), 3.56 (t, J = 8 Hz, 2H, CH_2), 3.44 (t, J = 8 Hz, 2H, CH_2), 3.23 (s, 9H, CH_3), 2.53 (s, 6H, CH_3), 2.43 (s, 6H, CH_3). ^{13}C NMR (100 MHz, d^6-DMSO), δ: 154.7, 141.4, 138.4, 131.1, 122.4, 63.8, 52.3, 22.0, 16.4, 14.2. TOF MS: m/z calcd for $C_{18}H_{27}BF_2N_3^+$ $[M]^+$ 334.2261, found: 334.2283.

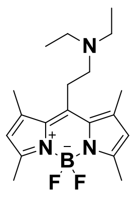

5

Diethylamine (0.65 mL, 6.28 mmol) was added to a solution of compound **3**[8] (82 mg, 0.314 mmol) in dry CH_2Cl_2 (4.0 mL) in a reaction vial. After the reaction mixture was refluxed for 3 d, the resulting products were diluted with CH_2Cl_2 and washed with sat. NH_4Cl solution and brine. The organic layer was dried ($MgSO_4$) and concentrated under reduced pressure. The residue was purified by column chromatography (silica, CH_2Cl_2/MeOH = 99/1, v/v) to obtain compound **5** (75.3 mg, 69.3 %). ^{1}H NMR (100 MHz, $CDCl_3$), δ: 6.05 (s, 2H, pyrrole-H), 3.19 (t, J = 4 Hz, 2H, CH_2), 2.66 (t, J = 4 Hz, 2H, CH_2), 2.60 (q, J = 8 Hz, 4H, CH_2), 2.51 (s, 6H, CH_3), 2.47 (s, 6H, CH_3), 1.04 (t, J = 8 Hz, 6H, CH_3). ^{13}C NMR (100 MHz, $CDCl_3$), δ: 154.0, 140.5, 137.5, 131.7, 121.7, 55.2, 46.7, 27.8, 16.6, 14.5, 11.6. TOF MS: m/z calcd for $C_{19}H_{29}BF_2N_3^+$ $[M+H]^+$ 348.2417, found: 348.2428.

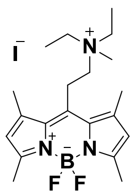

BP-3

Compound **5** (0.14 mmol, 50 mg) was dissolved in acetone (3 mL). Methyl iodide (0.43 mmol, 0.03 mL) was added dropwise to the above solution, and the mixture was stirred at 25°C under N_2 atmosphere for 2 h. The solid product **BP-3** (38.0 mg, 54.0 %) was obtained by filtration and being washed with acetone. ^{1}H NMR (400 MHz, d^6-DMSO), δ: 6.31 (s, 2H, pyrrole-H), 3.48 (m, 8H, CH_2), 3.09 (s, 3H, CH_3), 2.51 (s, 6H, CH_3), 2.42 (s, 6H, CH_3), 1.25 (t, J = 8 Hz, 6H, CH_3). ^{13}C NMR (100 MHz, d^6-DMSO), δ: 155.2, 141.8, 139.3, 131.8, 123.0, 63.8, 56.2, 22.1, 16.9, 14.7, 8.2. TOF MS: m/z calcd for

$C_{20}H_{31}BF_2N_3^+$ $[M]^+$ 362.2574, found: 362.2570.

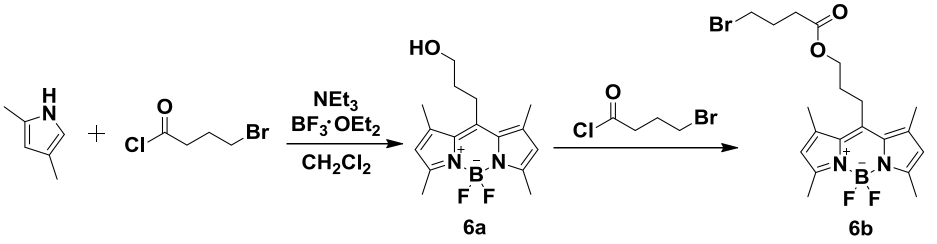

2,4-Dimethylpyrrole (2.0 mL, 19 mmol) was added to a solution of 4-bromobutanoyl chloride (1.0 mL, 8.8 mmol) in CH_2Cl_2 (20 mL) over 10 min at 0 °C. The reaction mixture was stirred at 0 °C for 30 min, then warmed up to 25 °C and stirred for an additional 30 min. Et_3N (3.7 mL, 26 mmol) was then added in small portions at 0 °C and the mixture was stirred at 25 °C for 10 min. BF_3•OEt_2 (5.5 mL, 44 mmol) was then added in portions and the mixture was stirred at 25 °C overnight. CH_2Cl_2 was removed under reduced pressure by rotary evaporation. The residue was purified by column chromatography on silica gel to obtain compound **6a** (546.0 mg, 20.2 %, *n*-hexane/EA = 1/1, v/v) and **6b** (423 mg, 31.4%, *n*-hexane/EA = 1/1, v/v). **6a** ^{1}H NMR (400 MHz, $CDCl_3$), δ: 6.05 (s, 2H, pyrrole-H), 3.80 (t, J = 4 Hz, 2H, CH_2), 3.06 (t, J = 4 Hz, 2H, CH_2), 2.52 (s, 6H, CH_3), 2.43 (s, 6H, CH_3), 1.86 (m, 2H, CH_2). ^{13}C NMR (100 MHz, $CDCl_3$), δ: 154.0, 146.0, 140.4, 131.5, 121.7, 62.5, 34.4, 25.0, 16.5, 14.4. TOF MS: m/z calcd for $C_{16}H_{22}BF_2N_2O^+$ $[M+H]^+$ 307.1788, found: 307.1790. **6b** ^{1}H NMR (400 MHz, $CDCl_3$), δ: 6.06 (s, 2H, pyrrole-H), 4.24 (t, J = 4 Hz, 2H, CH_2), 3.48 (t, J = 4 Hz, 2H, CH_2), 3.04 (t, J = 4 Hz, 2H, CH_2), 2.53 (t, J = 4 Hz, 2H, CH_2), 2.52 (s, 6H, CH_3), 2.43 (s, 6H, CH_3), 2.18 (m, 2H, CH_2), 1.97 (m, 2H, CH_2). ^{13}C NMR (100 MHz, $CDCl_3$), δ: 167.2, 149.1, 139.5, 135.0, 126.1, 116.6, 58.8, 27.3, 27.0, 25.4, 22.4, 19.8, 11.1, 9.2. TOF MS: m/z calcd for $C_{20}H_{27}BBrF_2N_2O_2^+$ $[M+H]^+$ 455.1312, found: 455.1324.

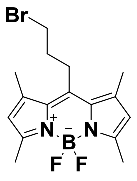

7

Compound **6a** (61.2 mg, 0.2 mmol) was dissolved in 10 mL 1,2-dichloroethane. PBr_3 (1.0 mL) was added dropwise to the above solution in ice bath. After stirring for 4 h at 45 °C, the reaction mixture was poured into sat. $NaHCO_3$ solution. The organic layer was dried over $MgSO_4$ and concentrated under reduced pressure. The residue was purified by column chromatography (silica, petroleum ether/EA = 4/1, v/v) to obtain compound **7** (63.5 mg, 86.3 %). ^{1}H NMR (400 MHz, $CDCl_3$), δ: 6.06 (s, 2H, pyrrole-H), 3.56 (t, J = 4 Hz, 2H, CH_2), 3.13 (t, J = 4 Hz, 2H, CH_2), 2.52 (s, 6H, CH_3), 2.45 (s, 6H, CH_3), 2.17 (m, 2H, CH_2). ^{13}C NMR (100 MHz, $CDCl_3$), δ: 154.4, 144.2, 140.3, 131.5, 121.9, 34.0, 32.8, 27.2, 16.7, 14.5. TOF MS: m/z calcd for $C_{16}H_{20}BBrF_2N_2^+$ $[M]^+$ 368.0871, found: 368.0878.

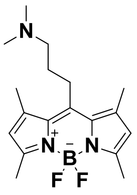

8

Compound **7** (37.0 mg, 0.1 mmol), dimethylamine (0.10 mL, 2 M in THF, 0.2 mmol), and potassium carbonate (28.0 mg, 0.2 mmol) were dissolved in 5 mL acetonitrile. The mixture was refluxed for 3 h under N_2 atmosphere. After filtration, the solvent was removed under reduced pressure by rotary evaporation. The crude product was purified by column chromatography (silica, CH_2Cl_2/MeOH = 200/10, v/v) to obtain compound **8** (18.0 mg, 54.0 %). ^{1}H NMR (400 MHz, $CDCl_3$), δ: 6.07 (s, 2H, pyrrole-H), 3.03 (t, J = 4 Hz, 2H, CH_2), 2.84 (t, J = 4 Hz, 2H, CH_2), 2.63 (s, 6H, CH_3), 2.51 (s, 6H, CH_3), 2.42 (s, 6H, CH_3), 2.04 (m, 2H, CH_2). ^{13}C NMR (100 MHz, $CDCl_3$), δ: 154.3, 144.7, 140.2, 131.3, 121.9, 57.5, 43.7, 31.0, 25.0, 16.4, 14.4. TOF MS: m/z calcd for $C_{18}H_{27}BF_2N_3^+$ $[M+H]^+$ 334.2261, found: 334.2259.

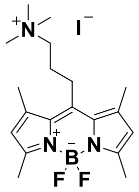

BP-4

Methyl iodide (0.9 mmol, 0.056 mL) was added dropwise to the solution of compound **8** (0.15 mmol, 50 mg) in acetone (3 mL). Then the mixture was stirred at 25 °C under N_2 atmosphere for 4 h, and the solid product **BP-4** (32.0 mg, 44.9%) was obtained by filtration and being washed with acetone. ^{1}H NMR (400 MHz, d^{6}-DMSO), *δ*: 6.26 (s, 2H, pyrrole-H), 3.45 (t, *J* = 4 Hz, 2H, CH_2), 3.30 (t, *J* = 4 Hz, 2H, CH_2), 3.07 (s, 9H, CH_3), 2.45 (s, 6H, CH_3), 2.41 (s, 6H, CH_3), 1.94 (m, 2H, CH_2). ^{13}C NMR (100 MHz, d^{6}-DMSO), *δ*: 153.4, 145.6, 140.9, 130.7, 121.8, 63.8, 52.2, 30.1, 24.5, 15.9, 14.1. TOF MS: m/z calcd for $C_{19}H_{29}BF_2N_3^+$ $[M]^+$ 348.2417, found: 348.2424.

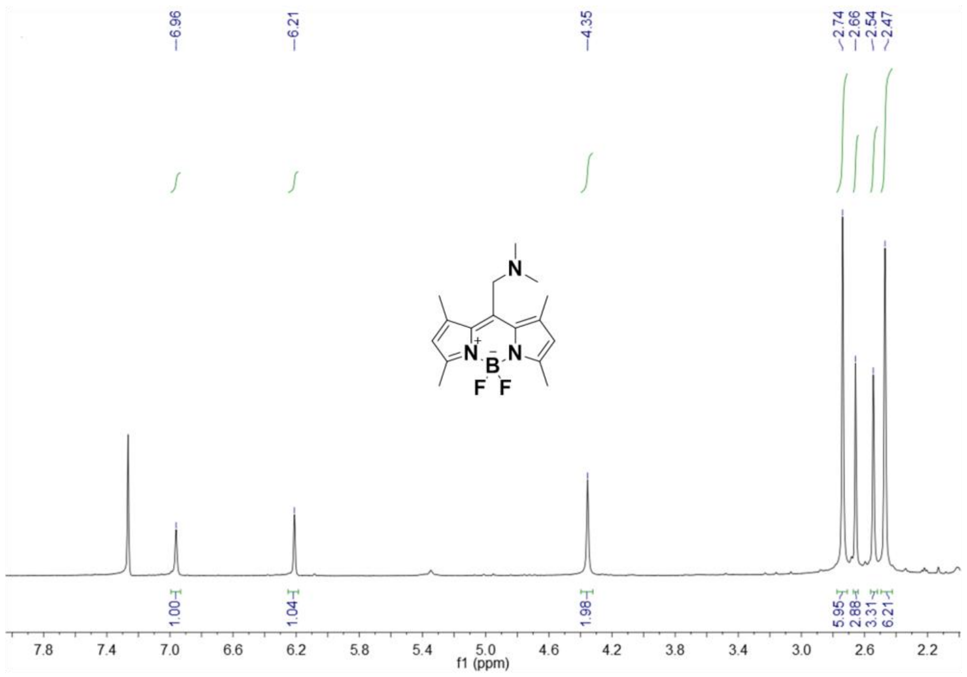

Supplementary Figure S12. ^{1}H-NMR of compound **2**

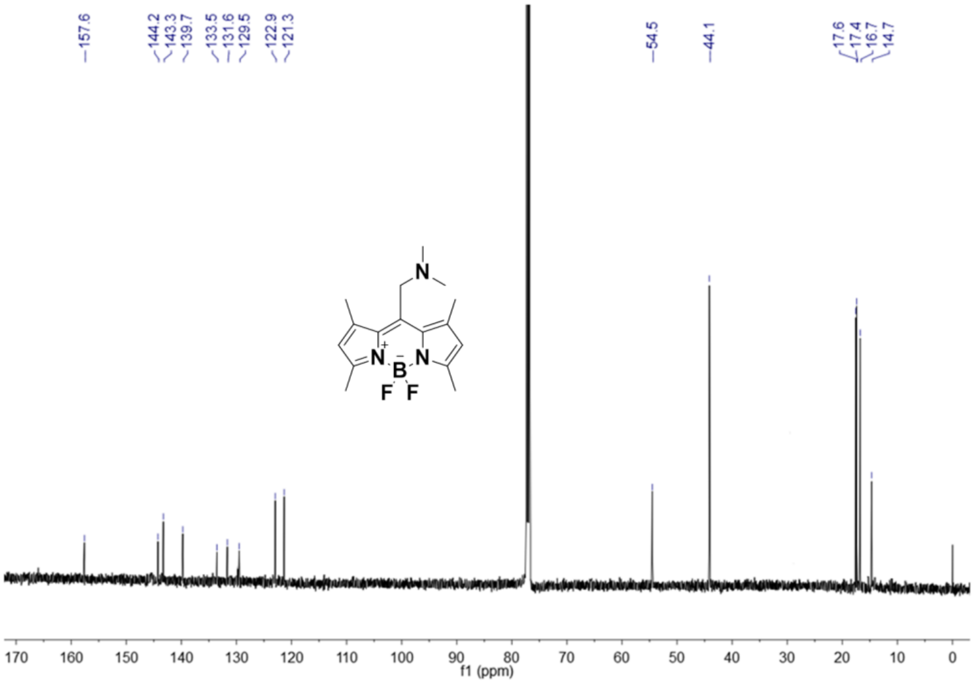

Supplementary Figure S13. ^{13}C-NMR of compound **2**

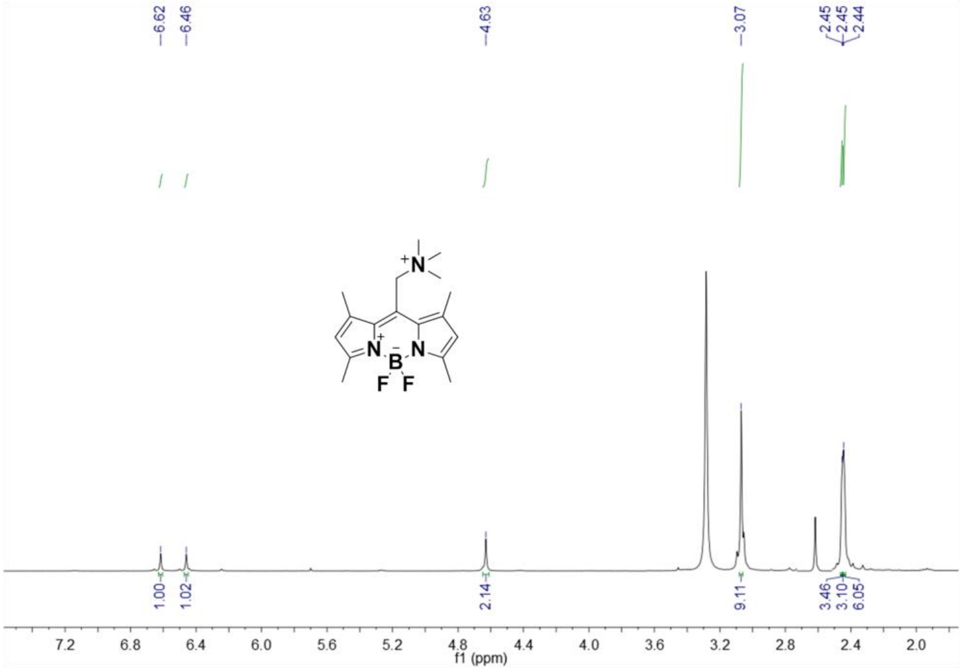

Supplementary Figure S14. ^{1}H-NMR of **BP-1**

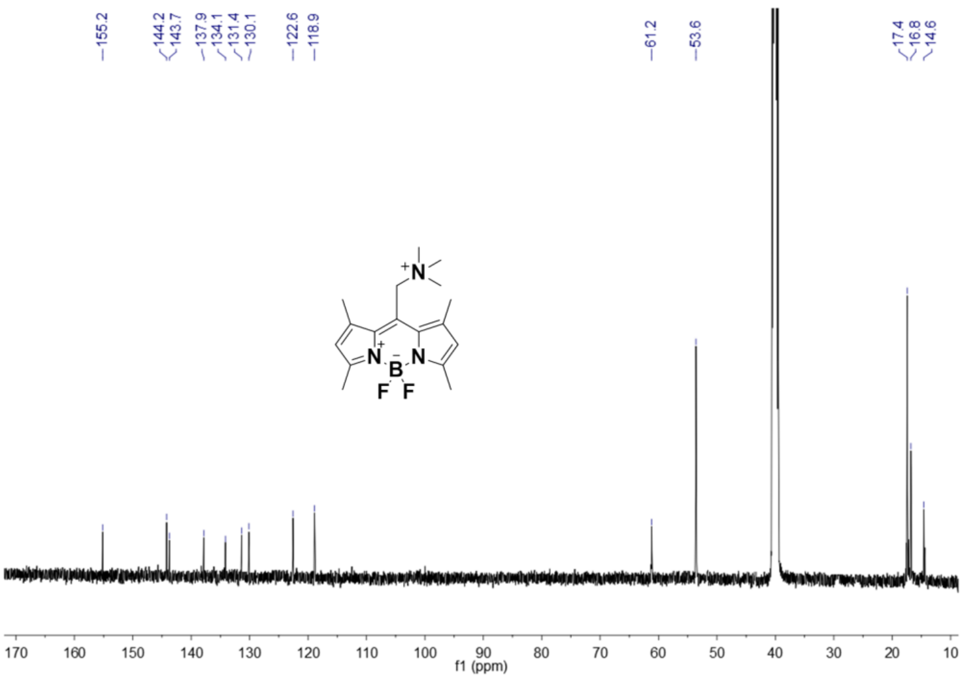

Supplementary Figure S15. ^{13}C-NMR of **BP-1**

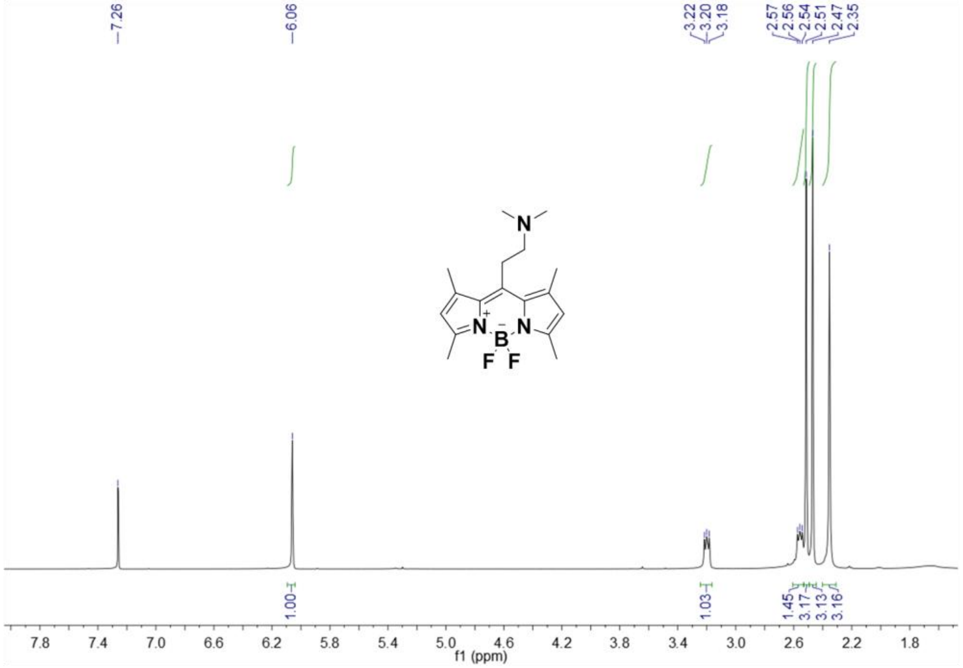

Supplementary Figure S16. ^{1}H-NMR of compound **4**

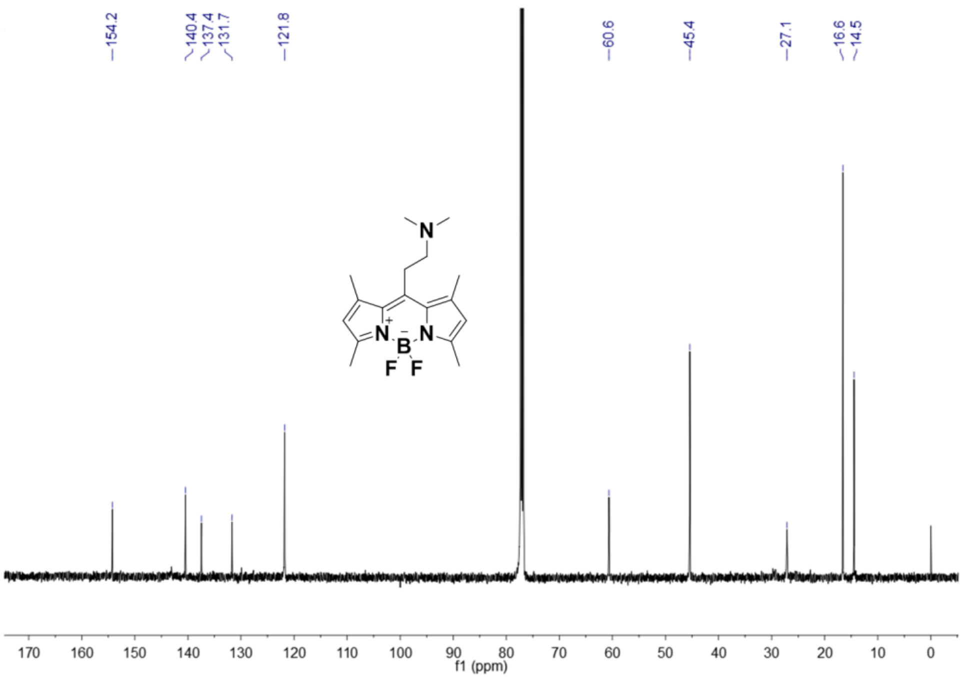

Supplementary Figure S17. ^{13}C-NMR of compound **4**

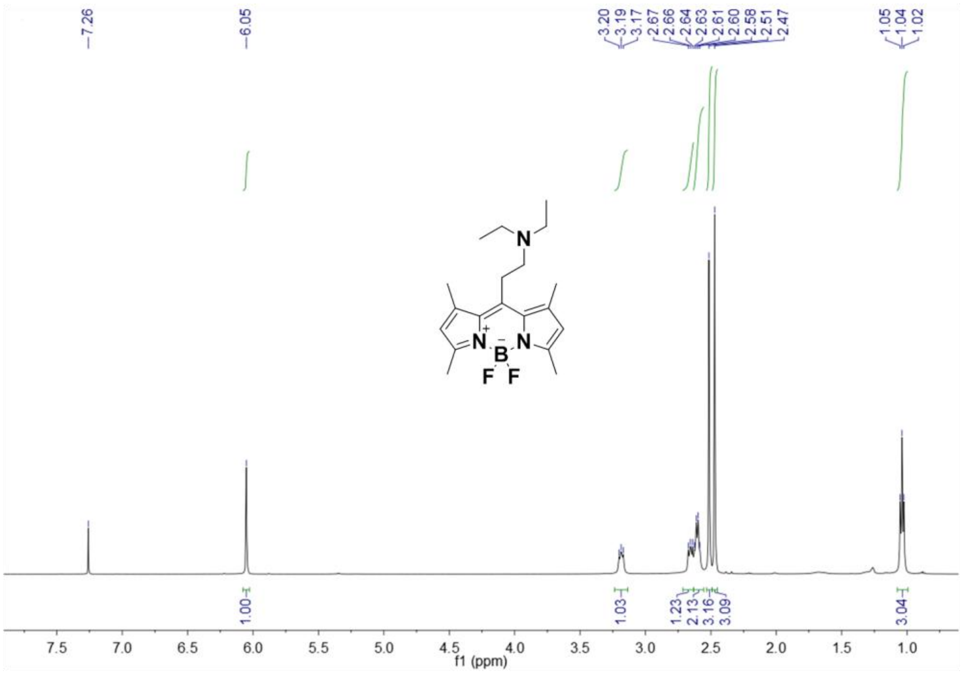

Supplementary Figure S18. ^{1}H-NMR of compound **5**

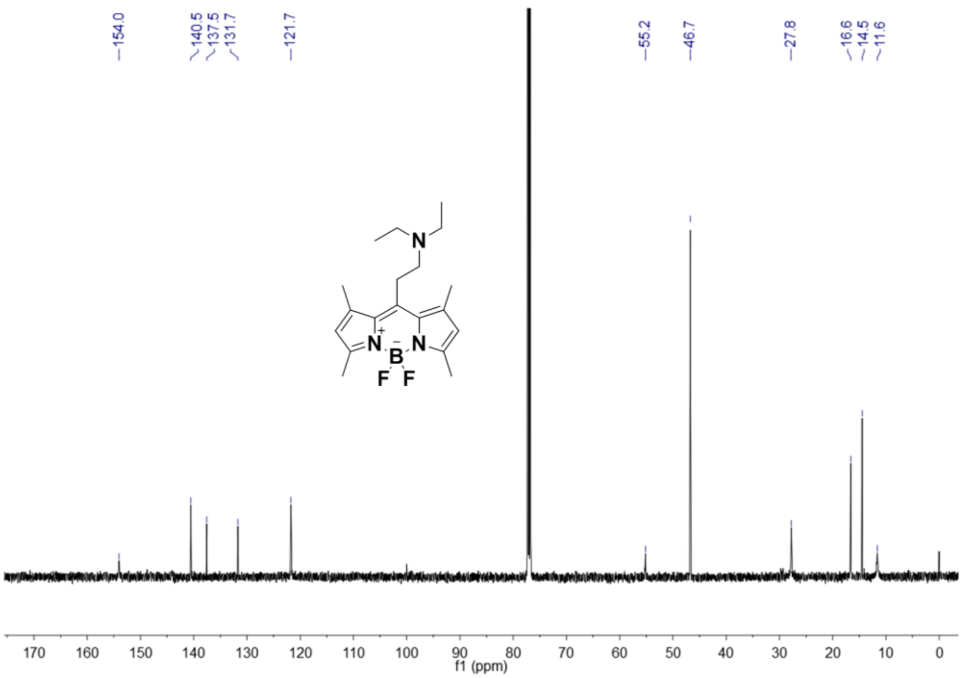

Supplementary Figure S19. ^{13}C-NMR of compound **5**

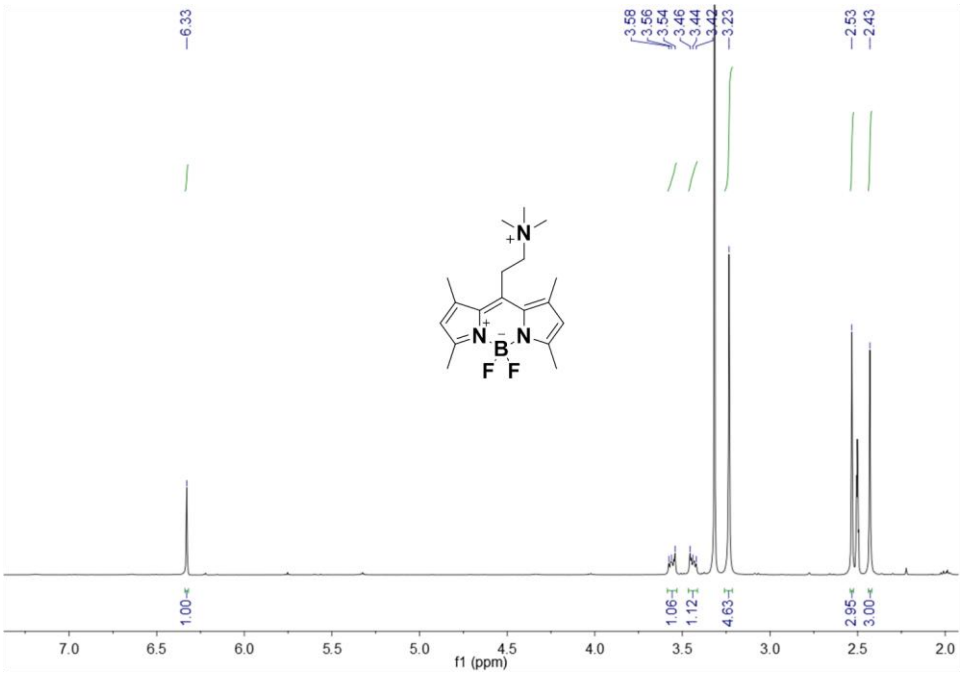

Supplementary Figure S20. ^{1}H-NMR of **BP-2**

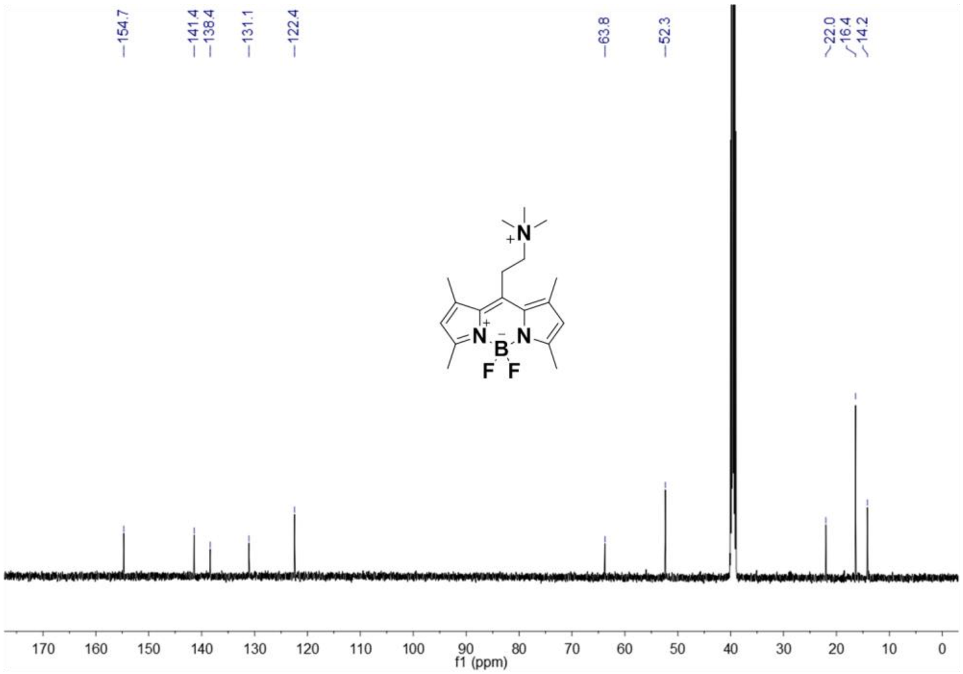

Supplementary Figure S21. ^{13}C-NMR of **BP-2**

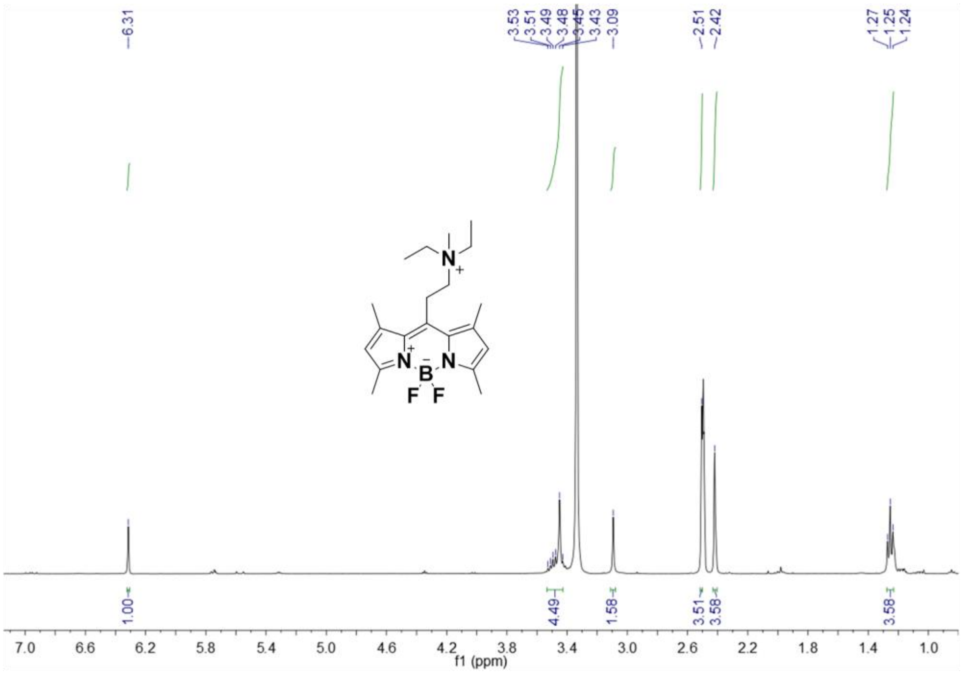

Supplementary Figure S22. ^{1}H-NMR of **BP-3**

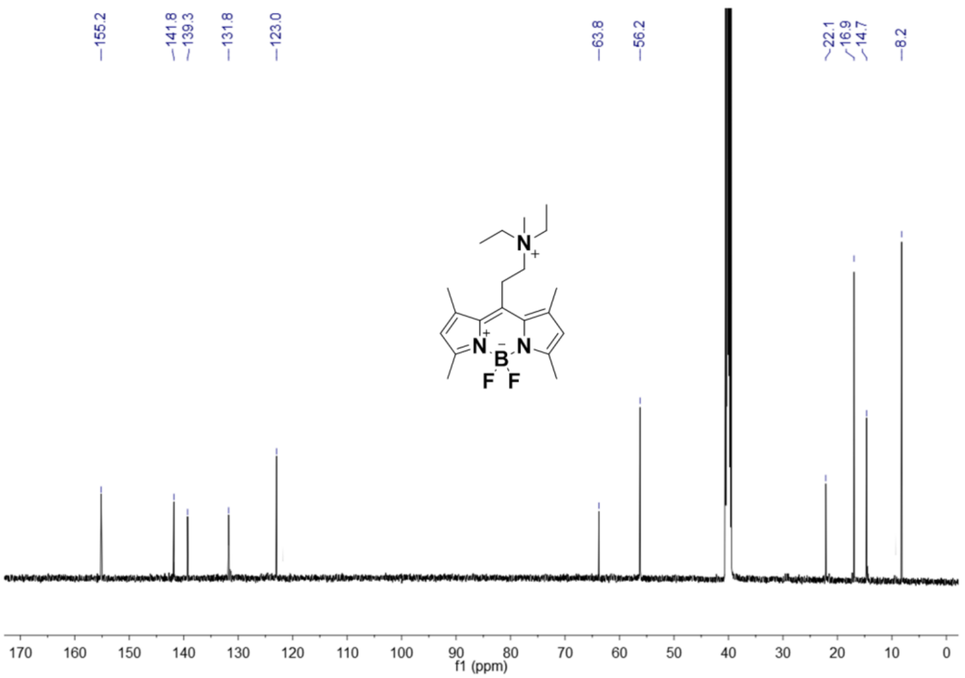

Supplementary Figure S23. ^{13}C-NMR of **BP-3**

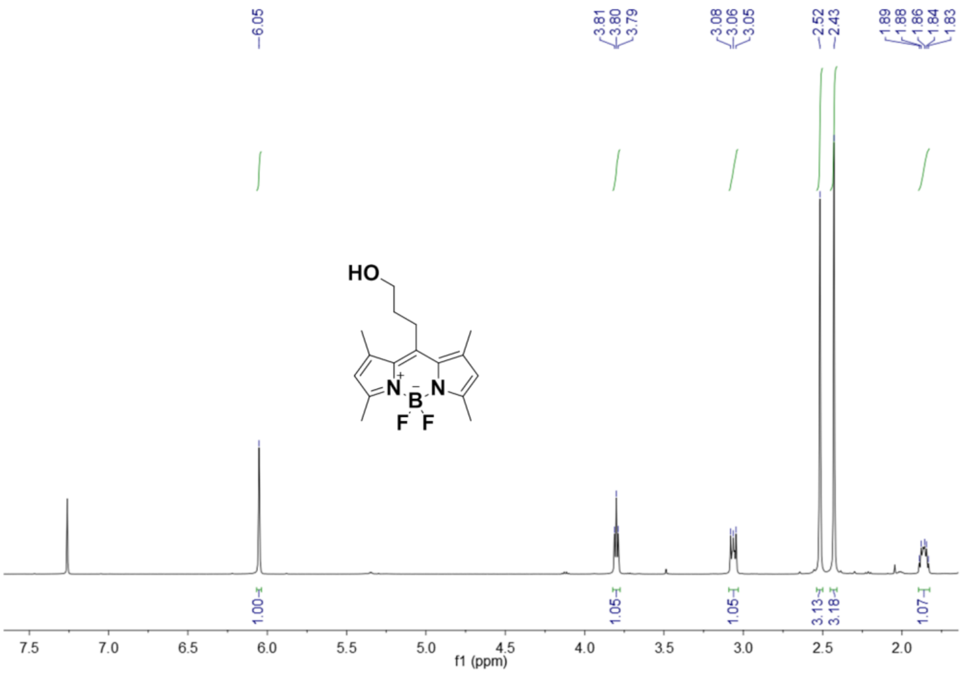

Supplementary Figure S24. ^{1}H-NMR of compound **6a**

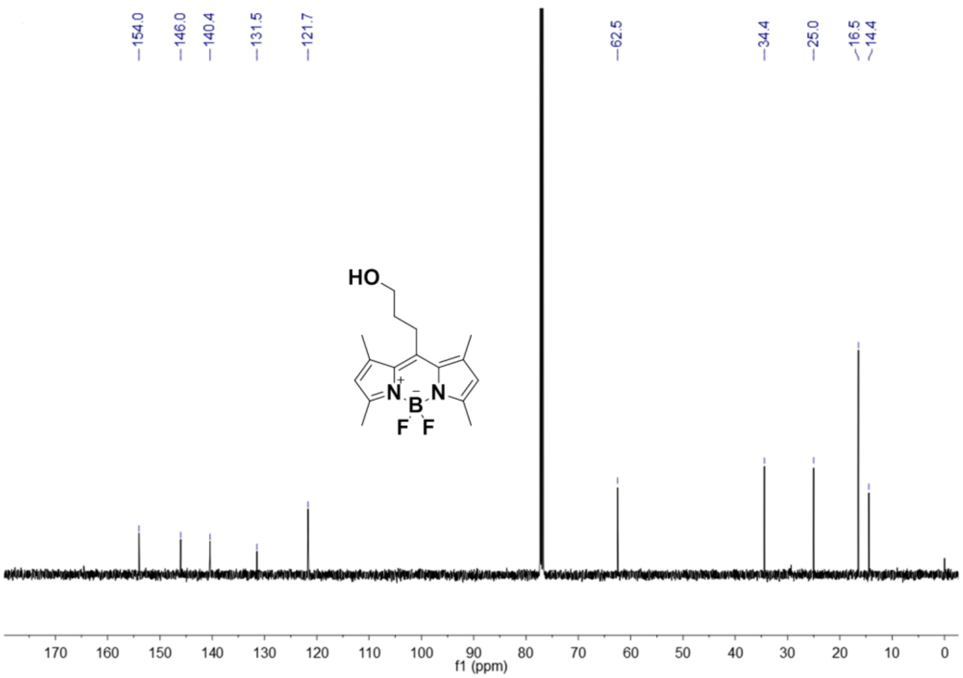

Supplementary Figure S25. ^{13}C-NMR of compound **6a**

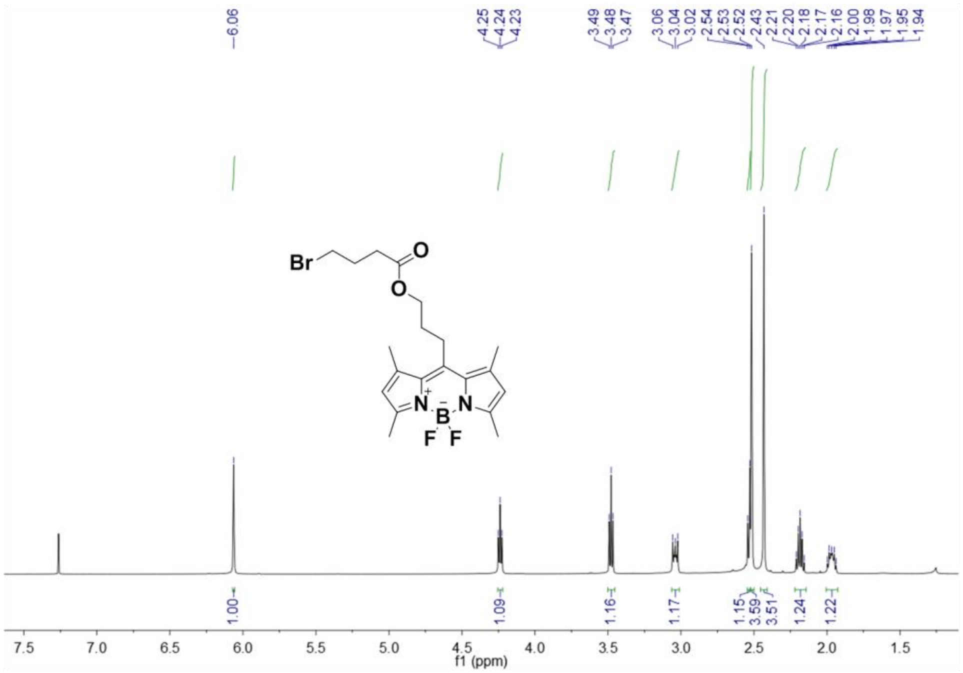

Supplementary Figure S26. ^{1}H-NMR of compound **6b**

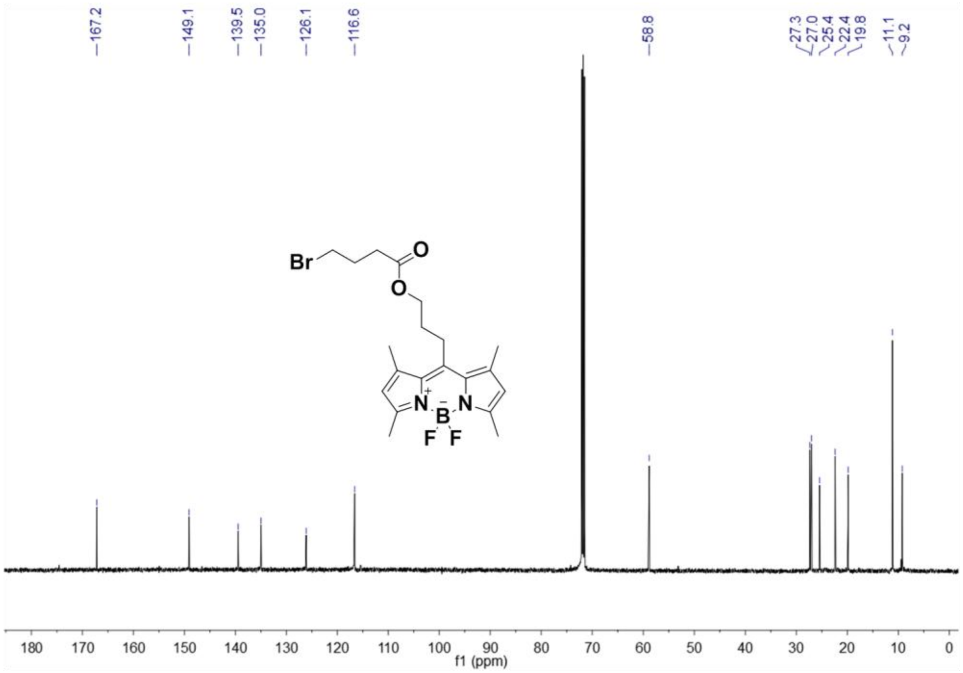

Supplementary Figure S27. ^{13}C-NMR of compound **6b**

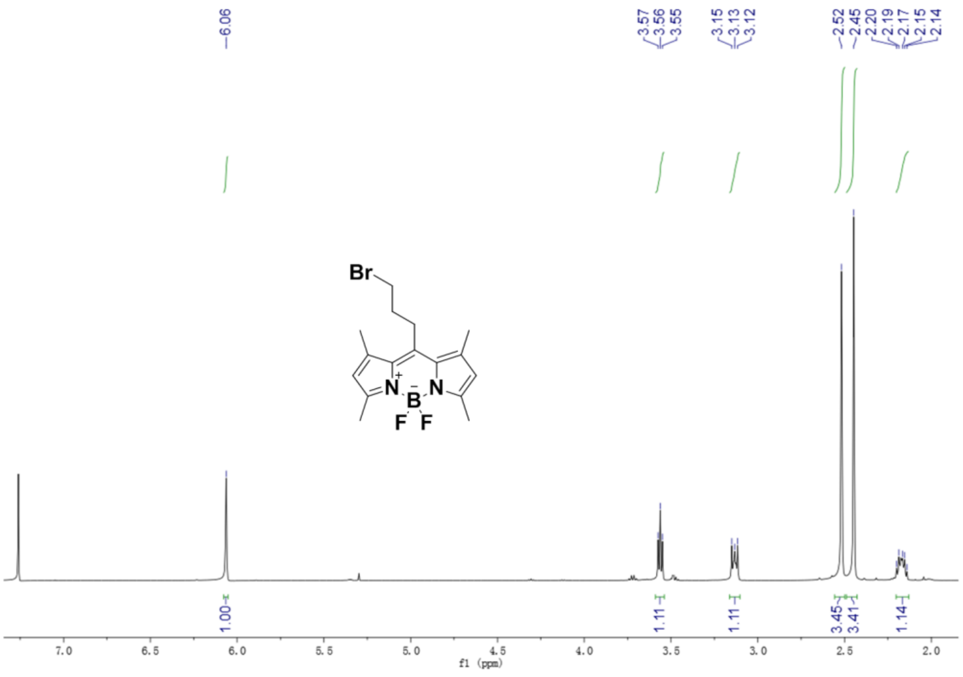

Supplementary Figure S28. ^{1}H-NMR of compound **7**

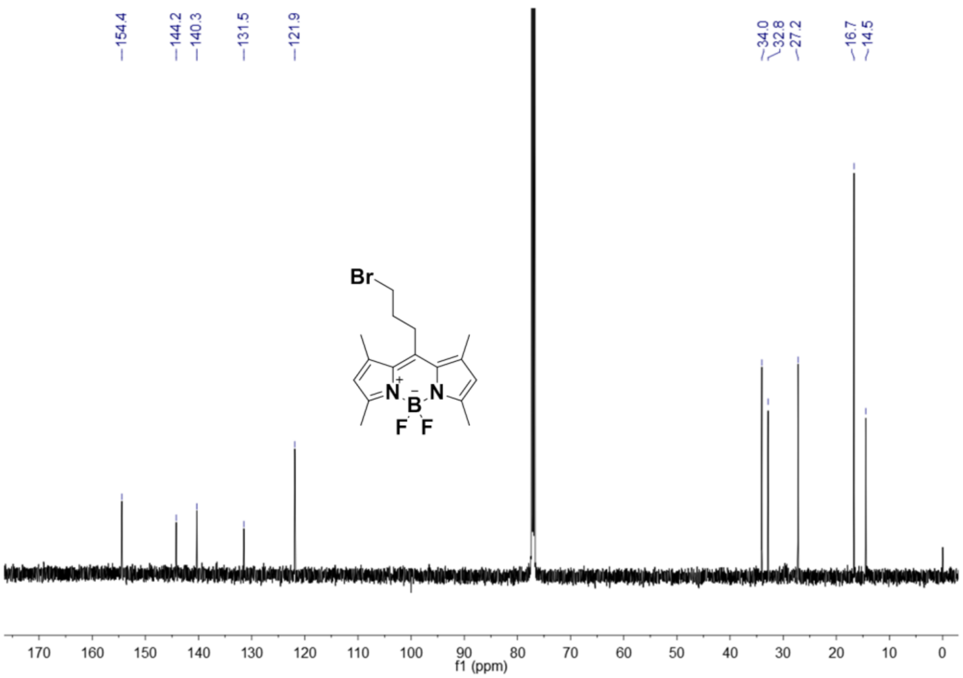

Supplementary Figure S29. ^{13}C-NMR of compound **7**

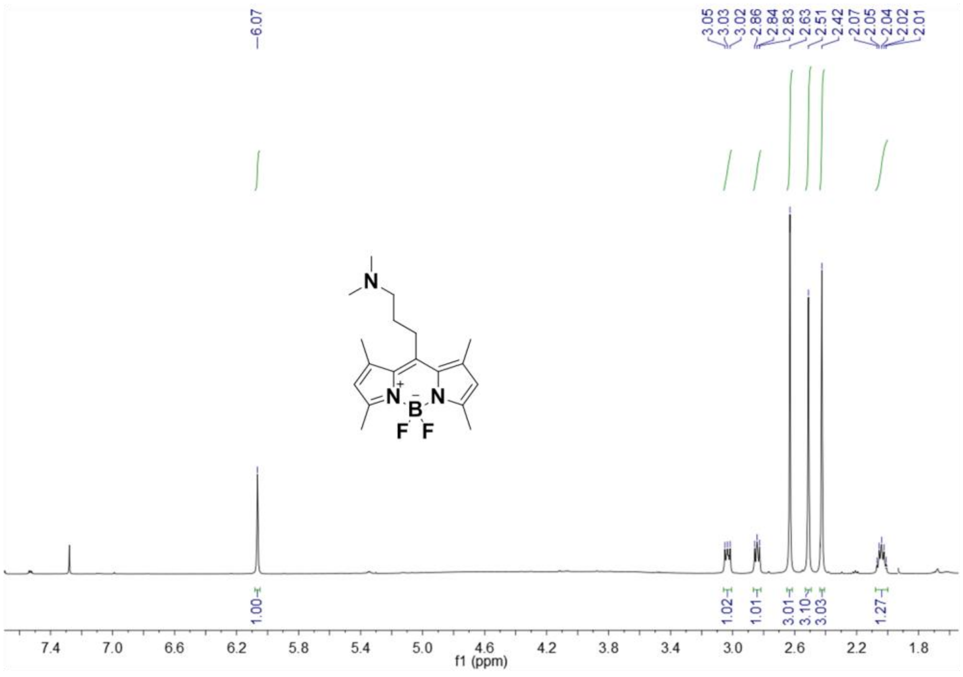

Supplementary Figure S30. ^{1}H-NMR of compound **8**

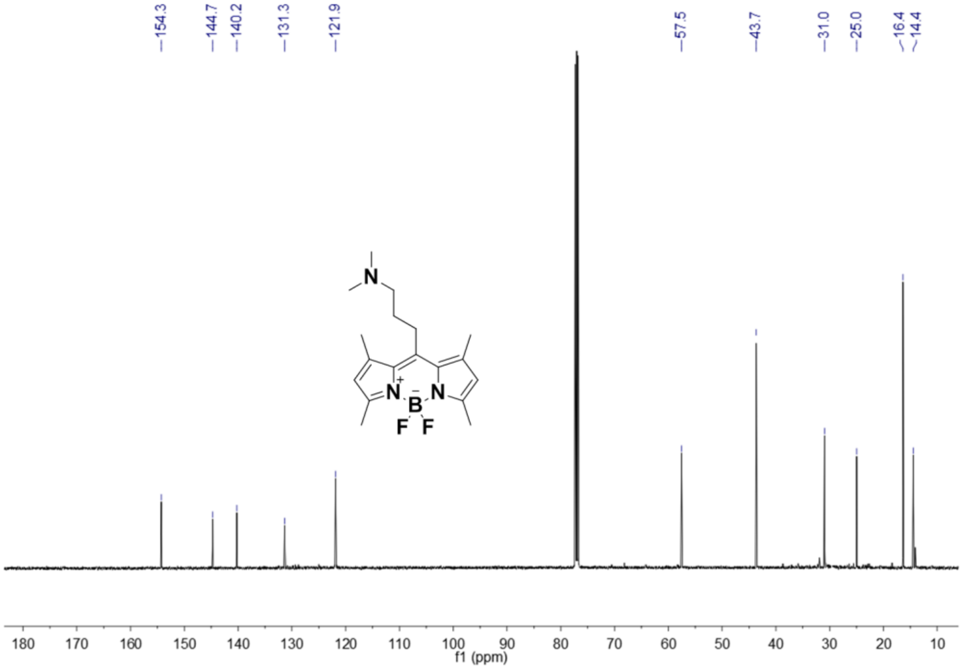

Supplementary Figure S31. ^{13}C-NMR of compound **8**

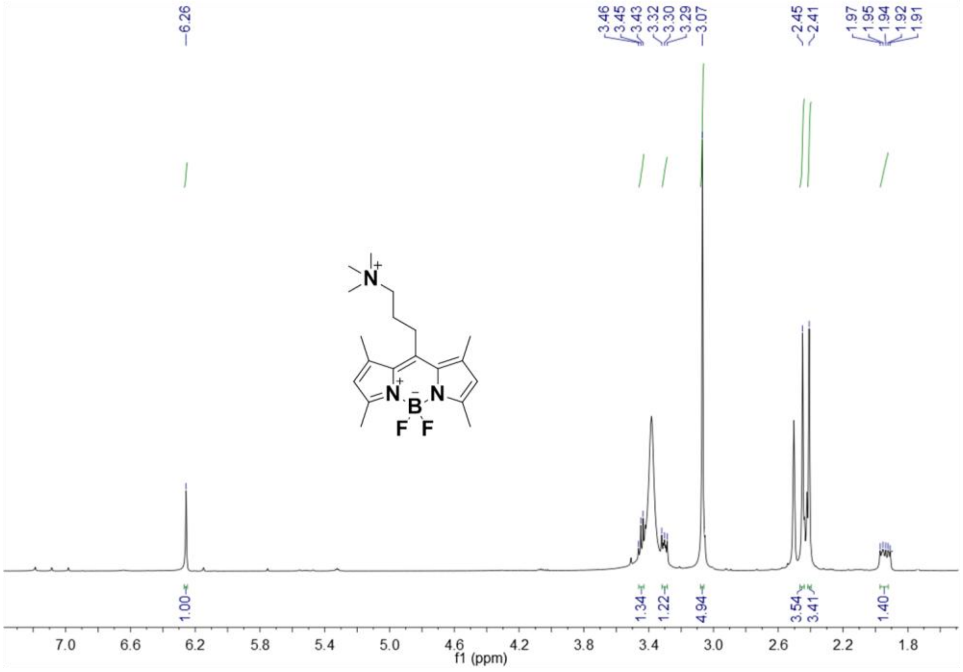

Supplementary Figure S32. ^{1}H-NMR of **BP-4**

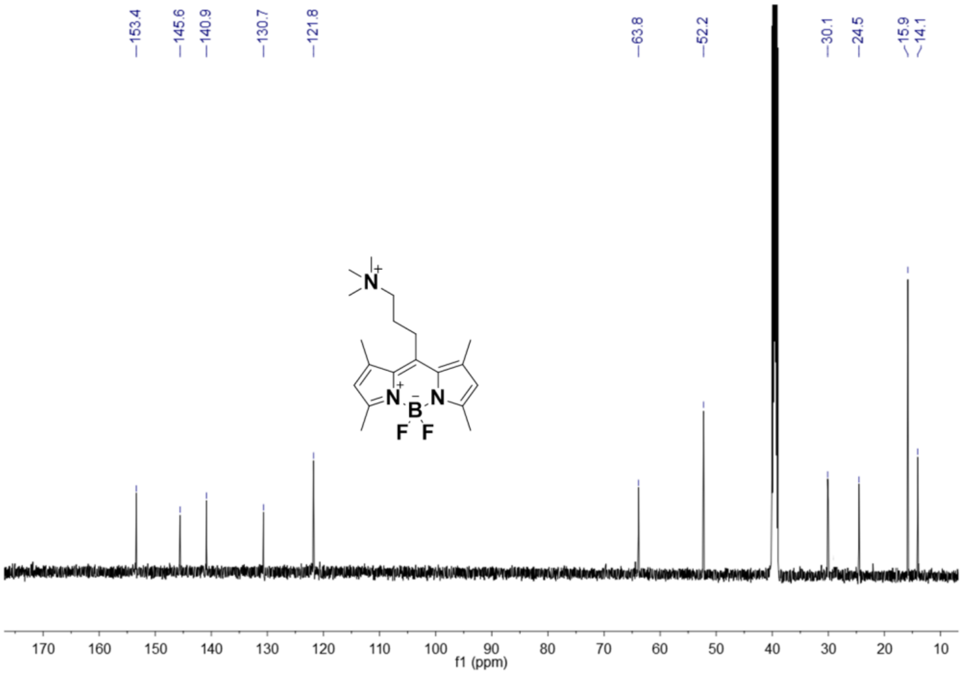

Supplementary Figure S33. ^{13}C-NMR of **BP-4**

Reference

1 Firisch, M. J. *et al. Gaussian 09, Revision A02* (Gaussian Inc., 2009).

2 Zhou, L.-C. *et al.* The charge transfer mechanism and spectral properties of a near-infrared heptamethine cyanine dye in alcoholic and aprotic solvents. *J. Photochem. Photobiol. A* **187**, 305-310 (2007).

3 Cao, J. *et al.* The nature of the different environmental sensitivity of symmetrical and unsymmetrical cyanine dyes: an experimental and theoretical study. *Phys. Chem. Chem. Phys.* **14**, 13702-13708 (2012).

4 Sholl, D. & Steckel, J. A. *Density Functional Theory* (Wiley-interscience, 1990).

5 Karstens, T. & Kobs, K. Rhodamine B and rhodamine 101 as reference substances for fluorescence quantum yield measurements. *J. Phys. Chem.* **84**, 1871-1872 (1980).

6 Casey, K. G. & Quitevis, E. L. Effect of solvent polarity on nonradiative processes in xanthene dyes: Rhodamine B in

normal alcohols. *J. Phys. Chem.* **92**, 6590-6594 (1988).

7 Wu, Y. *et al.* Boron dipyrromethene fluorophore based fluorescence sensor for the selective imaging of Zn(ii) in living cells. *Org. Biomol. Chem.* **3**, 1387-1392 (2005).

8 Guo, B. *et al.* Synthesis and spectral properties of new boron dipyrromethene dyes. *Dyes Pigm.* **73**, 206-210 (2006).
